# A single‐view‐based electroacoustic tomography imaging using deep learning for electroporation monitoring

**DOI:** 10.1002/mp.70623

**Published:** 2026-08-10

**Authors:** Jie Zhang, Yifei Xu, Leshan Sun, Yankun Lang, Liangzhong Xiang, Lei Ren

**Affiliations:** ^1^ Department of Radiation Oncology University of Maryland School of Medicine Baltimore Maryland USA; ^2^ Department of Biomedical Engineering University of California Irvine California USA

**Keywords:** deep‐learning extrapolation model, electroacoustic tomography, electroporation, limited‐view reconstruction, ultrasound imaging

## Abstract

**Background:**

Electroacoustic tomography (EAT) is an emerging imaging technique with potential for guiding electroporation therapy. However, in clinical applications, EAT scan is typically limited to a single view acquisition, leading to severe artifacts in the reconstruction.

**Purpose:**

This study aims to develop novel deep‐learning models to substantially enhance the image quality of the single‐view EAT reconstruction.

**Methods:**

We proposed a two‐stage deep learning model (EXPP‐Net) to address the limited view problem in EAT image reconstruction. The two‐stage model comprises extrapolation and post‐processing sub‐models. The extrapolation sub‐model receives a single‐view sinogram ***u*** *_α_* acquired at any angle *α* and a rotation angle *γ*∈[−60°, −24°, 24°, 60°], and predicts four single‐view sinograms at angles *α*+*γ*. The 5 sinograms (i.e., ***u*** *_α_*_‐60°_, ***u*** *_α_*_‐24°_, ***u*** *_α_*, ***u*** *_α_*_+24°_, ***u*** *_α_*_+60°_) are back‐projected to reconstruct an initial sparse‐view EAT image (***m***_sv_). The post‐processing sub‐model enhances ***m***_sv_’s quality to a full‐view image (***m***_fv_). These sub‐models were trained using experimental data. Using a linear‐array probe, 54 full‐view datasets (60 or 90 views each) were acquired by delivering electrical pulses via two tungsten electrodes rotated from −180° to 180° in a water tank. Each view ***u*** *_α_*, together with {***u*** *_α_*_‐60°_, ***u*** *_α_*_‐24°_, ***u*** *_α_*_+24°_, ***u*** *_α_*_+60°_} and ***m***_fv_, forms a view set for model training. Data splitting was performed at the dataset level, yielding 34/10/10 datasets for training/validation/test, corresponding to 2190/600/600 view sets. The model performance was evaluated using root mean square error (RMSE), structural similarity index measure (SSIM), and the iso‐pressure line Dice similarity coefficients (DICE). Statistical analysis used a linear mixed‐effects model with Benjamini–Hochberg FDR correction (*α *= 0.05), and effect size was defined as standardized fixed‐effect coefficients (*β*/*σ*). For comprehensive evaluation, the model was re‐trained and evaluated on convex‐array probe data, and further tested in vivo on one mouse. The model was also compared with a previously published method to demonstrate its advantages.

**Results:**

The quality of EAT images reconstructed from single‐view acquisition was substantially improved by EXPP‐Net, showing good agreement with full‐view images (linear‐array probe study: RMSE: 0.0033 ± 0.0023, SSIM: 0.9968 ± 0.0059, median DICE ≥ 0.9450; convex‐array probe study: RMSE: 0.0083 ± 0.0034, SSIM: 0.9958 ± 0.0038, median DICE ≥ 0.8837). In vivo results demonstrated substantial distortion correction. Comparison with the prior method on RMSE, SSIM, and DICE yielded large effect sizes (|*β*/*σ*| > 0.8), with all comparisons reaching statistical significance (*p* < 0.05).

**Conclusions:**

The proposed model generated full‐view EAT imaging from a single‐view sinogram, substantially improving the quality and precision of EAT. These results suggest its potential for real‐time electroporation verification.

## 1 INTRODUCTION

Electroporation is a technique which uses an external electrical field to increase the cell membrane permeability.^1^, ^2^ Electroporation can be classified into reversible electroporation (RE)^3^, ^4^ and irreversible electroporation (IRE). RE has been developed for electrochemotherapy to induce a transient increase in membrane permeability to promote the uptake of chemotherapeutic agents into tumor cells.^5^ IRE has been developed as an ablation therapy^6^, ^7^, ^8^ technique that triggers cell death by disrupting its membrane's physical structure. The efficacy of RE and IRE heavily depends on the accuracy of the electroporation delivery, therefore intraoperative monitoring of the deposited electronic energy and its spatial distribution from electroporation is critical to ensure its delivery precision and treatment efficacy.^6^

Electroacoustic tomography (EAT)^9^, ^10^, ^11^, ^12^ has been proposed as a novel imaging technique for electroporation verification. It is based on a physical phenomenon that the electron energy deposition can stimulate the emission of broadband ultrasound waves,^13^ which can be used for acoustic imaging to estimate the energy deposition. In 2023, Xu et al.^13^ used a ring‐array ultrasound transducer to capture the spatiotemporal dynamics of the local electric fields in a chicken breast, and first proved the potential of EAT for real‐time electroporation monitoring. Despite the promise of ring‐array ultrasound for EAT imaging, it is often inconvenient or even impractical to use in clinical scenarios. In contrast, a linear or convex array transducer is much more convenient and practical to use for EAT imaging.^14^ However, a major limitation of linear or convex array transducers is their single‐view limited‐angle acquisition, which leads to severe distortion in the reconstructed images.^15^ Although rotating the transducers around the patient can increase the scanning angle, it will inevitably slow down the scanning speed and prevent real‐time imaging for treatment verification.^14^ Addressing the image distortion in single‐view EAT imaging based on linear or convex array transducers is a critical need for electroporation verification to ensure the precision of the treatment.^15^

Deep learning has shown great potential for data processing,^16^, ^17^, ^18^, ^19^, ^20^, ^21^ image reconstruction^22^, ^23^, ^24^, ^25^ and image enhancement^26^, ^27^, ^28^, ^29^ to improve the quality of images reconstructed from undersampled data. One approach to address undersampling is to generate the missing data from the measured one by extrapolation.^30^, ^31^, ^32^ Wang et al.^33^ proposed a deep learning network to recover high‐quality computed tomography (CT) images with an extrapolation in sinogram domain. Their results showed that the network can achieve 30.40 dB of peak signal‐to‐noise ratio (PSNR) and 0.906 of structural similarity index measure (SSIM). However, this method's utility is limited to a specific scanning configuration. With the superior performance of generative adversarial networks (GANs) on image inpainting^34^, ^35^, ^36^, ^37^ (i.e., restoring an image's missing parts), Li et al.^38^ employed a sinogram‐inpainting‐GAN (SI‐GAN), consisting of a U‐Net generator and patch‐design discriminator, to repair the missing data and hence improve the sparse‐angle‐reconstructed image quality. Similar works have been reported by Podgorsak et al.’s^39^ and Wang et al.’s.^40^ The principle of these inpainting work is to learn the training samples’ probability distribution and restore the missing data by conforming them to the learned distribution.^38^, ^41^ However, no such technique has been developed for EAT imaging. EAT presents additional challenges with variations in the electroporation‐related signals due to changes in treatment parameters (e.g., the voltage, electrical pulse duration, etc.), making it difficult to build a model to be robust against different acquisitions.

Another approach to improve image quality is to perform image enhancement after the reconstruction.^15^, ^42^, ^43^, ^44^, ^45^, ^46^ Jiang et al.^15^ introduced a modified U‐net to enhance EAT from a single‐view image. Similarly, a three‐dimension U‐net was used to correct distortion in limited‐view proton‐acoustic imaging.^43^ However, the correction performance was limited because the model did not fully consider view‐angle‐dependent variations in distortion patterns. A review of the available literature indicates that studies specifically exploring high‐quality image generation from single‐view EAT acquisition remain limited.

**FIGURE 1:**
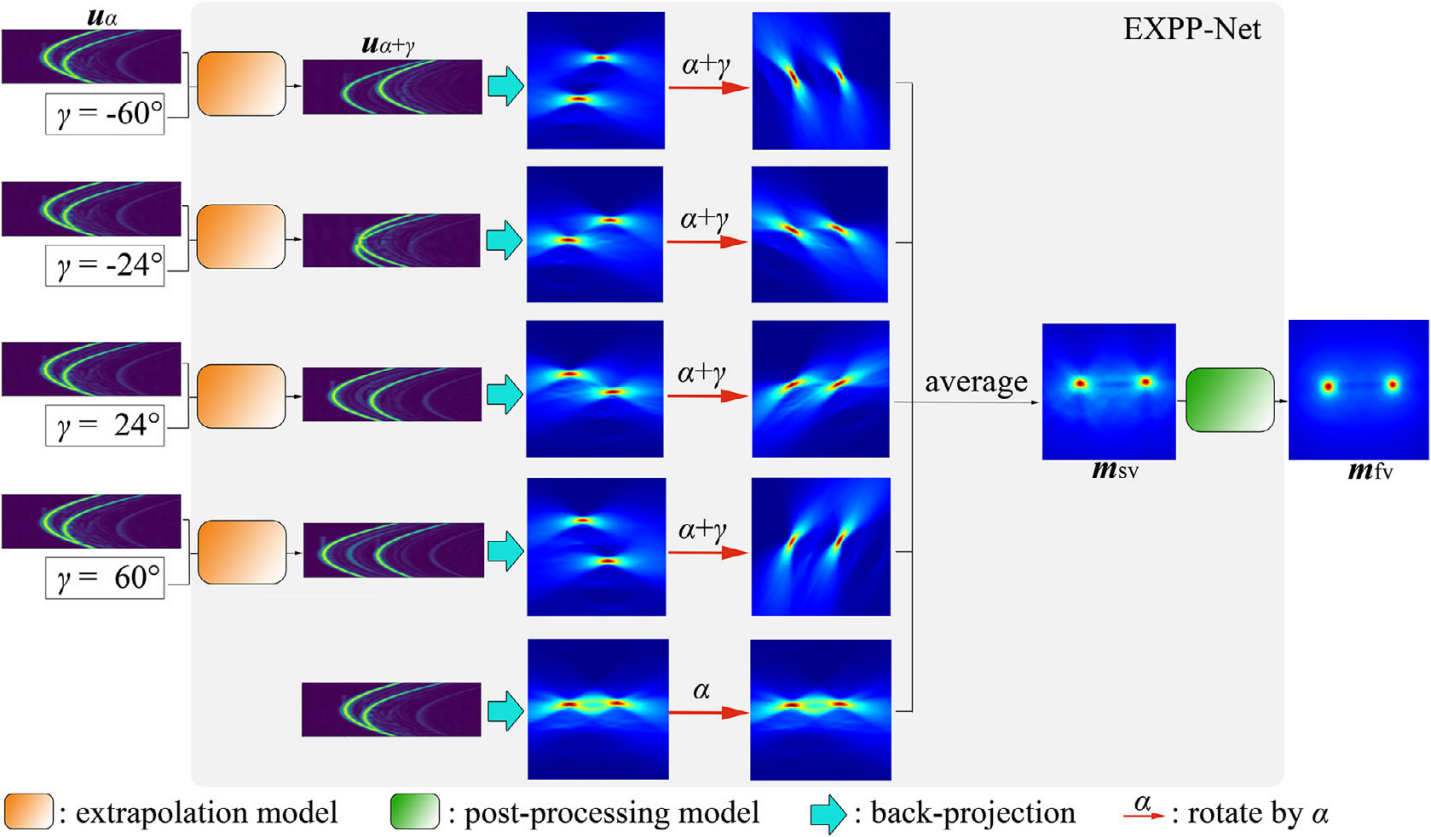
Illustration of our proposed EXPP‐Net.^a,b^. (a) Denotations: *γ*, rotation angle; *u_α_*, sinogram acquired at angle *α*; *u_α_*_+_ *_γ_*, extrapolated sinogram corresponding to *α*+*γ*; *m*_sv_, sparse‐view image; *m*_fv_, full‐view image. (b) Note that the four extrapolation models share the same model weights.

In this study, we propose a two‐stage cascaded deep learning model (as shown in Figure 1) for both sinogram extrapolation and image enhancement to generate a full‐view image from a single‐view sinogram for EAT imaging, where the variable definitions in this study are summarized in Table 1 and the methodological adjustments over the aforementioned approaches are provided in Supplementary Materials S1 (Table S1 and Figure S1). The first‐stage model is an extrapolation model in sinogram domain, that generates other 4 sinograms from the input single‐view one. The five sinograms are reconstructed into image domain using back projection algorithm,^47^ and then are transferred to the second‐stage model. The second‐stage model is a post‐processing model to correct the remaining image distortion. The two‐stage model is abbreviated as EXPP‐Net, since it consists of an EXtrapolation model and a Post‐Processing one. The contributions of our EXPP‐Net are:

(1). Two self‐designed convolutions (abbreviated as RotDRConv and RotTransDRConv), approximating the rotation‐induced signal variations, are employed in the extrapolation model.
(2). The EXPP‐Net is applicable for the single‐view signal acquired at any angle, making it applicable in different clinical scenarios.
(3). Compared to other EAT study on simulation data,^9^ the EXPP‐Net was trained and tested using the experiment data from scanning using both water and animals, further confirming its utility in real scenarios to substantially enhance the quality of EAT imaging for precision verification of electroporation treatments.

**TABLE 1:** Variable definitions.

Variable name	Description
*u_α_*	Sinogram acquired at angle *α*
*u_α_*_+_ *_γ_*	Extrapolated sinogram corresponding to *α*+*γ*
*Γ*	Rotation angle
*m*_sv_	Sparse‐view image
*m*_fv_	Full‐view image

The rest of this paper is organized as follows. Section 2 introduces our model's architecture and experiment settings. The EXPP‐Net was evaluated using experimental data in water tank and a mouse, and benchmarked against other published work for EAT image enhancement.^15^ The results are presented in section 3 and discussed in section 4. Section 5 concludes our work.

## 2 MATERIALS AND METHODS

### 2.1 Method overview

**FIGURE 2:**
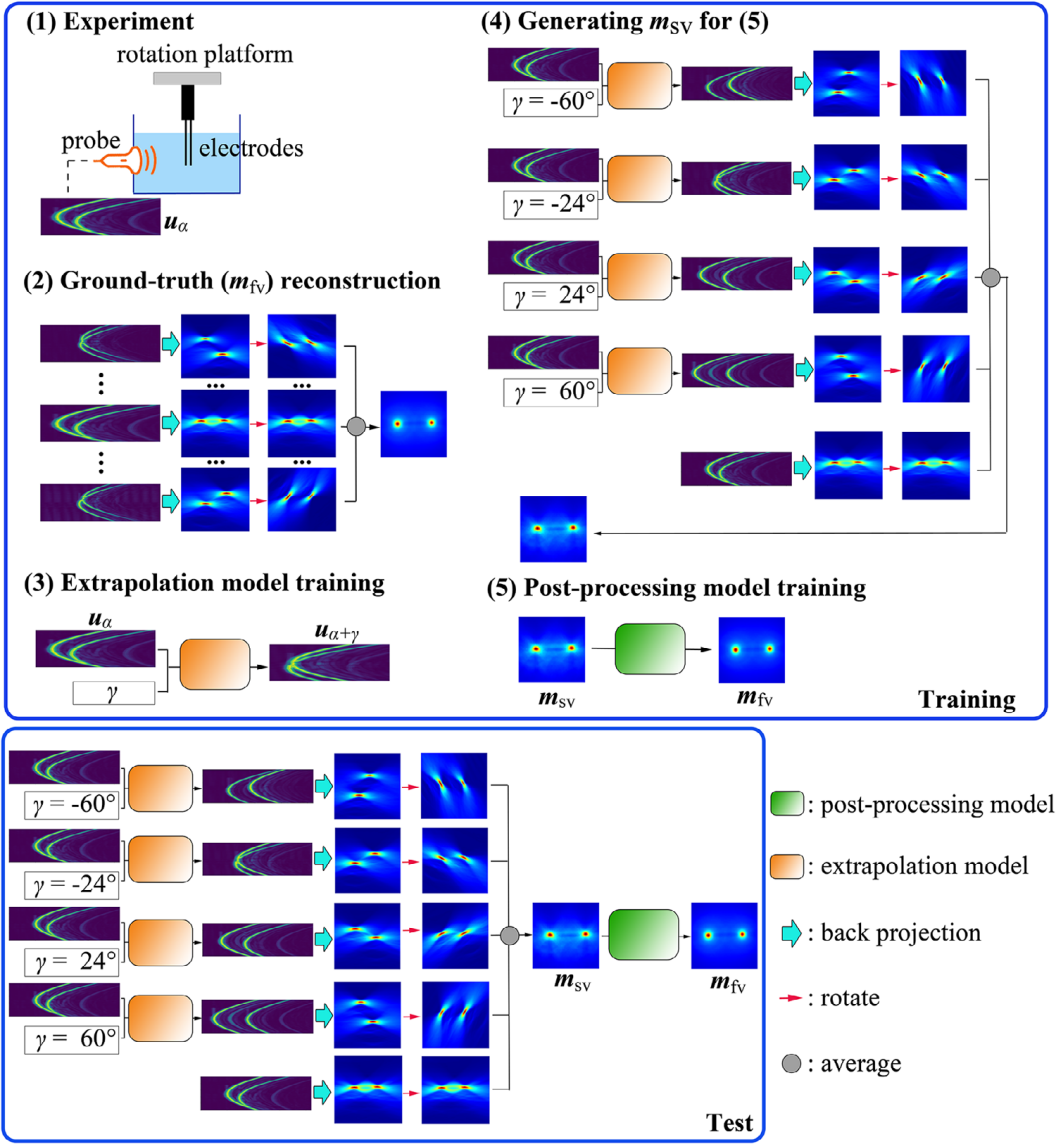
Schematic representation of our proposed EXPP‐Net.

Figure 2 shows a schematic representation of our work. The training framework is composed of the following blocks: (a) an extrapolation model that extrapolates multiple sinograms (***u*** *_α_*_+_ *_γ_*) according to its two input variables (i.e., the rotation angles *γ* and the sinogram ***u*** *_α_* acquired at angle *α*). An initial sparse view image (***m***_sv_) is reconstructed by the back‐projection method using both the extrapolated sinograms and the input one; (b) a post‐processing model that enhances the quality of the sparse‐view image (***m***_sv_) to a full‐view one (***m***_fv_). During test, the extrapolation model and the post‐processing model are cascaded to be our EXPP‐Net. In the following subsections, we detail the two models.

### 2.2 Network architectures

#### 2.2.1 The Extrapolation Model's Structure

**FIGURE 3:**
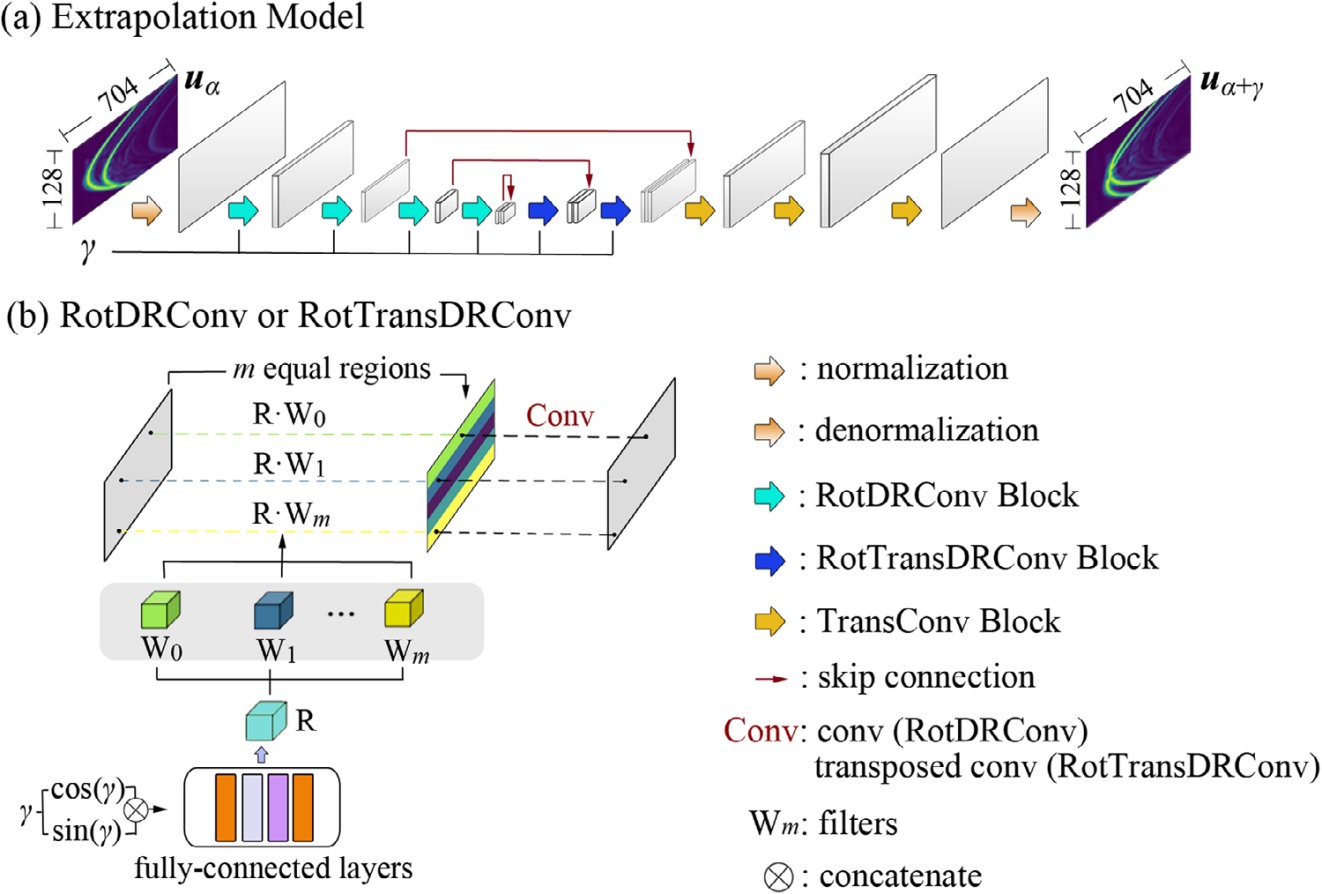
(a) Structure of the extrapolation model^a^ and (b) illustrations of RotDRConv and RotTransDRConv. ^a^RotDRConv Block: RotDRConv+LayerNorm+LeakyReLU; RotTransDRConv Block: RotTransDRConv+LayerNorm+LeakyReLU; TransConv Block: TransConv + LN + LeakyReLU (final: Tanh^1^). Other abbreviations are the same as those in Figures 1 and 2.

The extrapolation model's structure is shown in Figure 3(a). More details of the proposed network can be referred to in Supplementary Materials S2 (Figures S2 and S3). It employs a U‐net backbone. Each layer adopts large kernel size to fit with the signal's large spatial change caused by different rotation angles, since one axis of a sinogram refers to the distance between the ultrasound signal source and each transducer element. We used the layer normalization^48^ to ensure the model learns the consistent rotation‐induced signal change pattern, regardless of the input's distribution.

To approximate the signal changes caused by rotation, we propose two self‐designed convolutions (i.e., the RotDRConv and RotTransDRConv in Figure 3(b)) by modifying the dynamic region‐aware convolution (DRConv).^49^ Specifically, two regularities are observed in rotation‐induced signal changes: (a) at a fixed rotation angle, signals received by different transducer elements vary; (b) for a given transducer element, signal variations differ across rotation angles. To address regularity (a), RotDRConv and RotTransDRConv apply different filters (i.e., W_0_, W_1_, …, W*_m_* in Figure 3(b)) to different subregions of the input. To address regularity (b), fully connected layers generate modulation parameters (i.e., *R* in Figure 3(b)) that adjust the filter weights based on the rotation angle *γ*. More details can be found in Supplementary Materials S3(1‐2) (Figures S4 and S5).

The two input variables of the extrapolation model are ***u*** *_α_* (i.e., the sinogram acquired at the angle of *α*) and *γ* (i.e., the rotation angle), the output is an extrapolated sinogram ***u*** *_α_*_+_ *_γ_*. In our work, the *γ* is within a range of {−60°, −24°, 24°, 60°}. *γ* is constrained within (−90°, 90°) to ensure valid input to the convolution. This also enables the generation of four approximately evenly distributed extrapolated sinograms, avoiding single‐view distortion that would result from concentrated angles. A more detailed discussion of the choice of *γ* is provided in Supplementary Materials S3(3) (Figure S6).

#### 2.2.2 The Post‐processing Model's Structure

**FIGURE 4:**
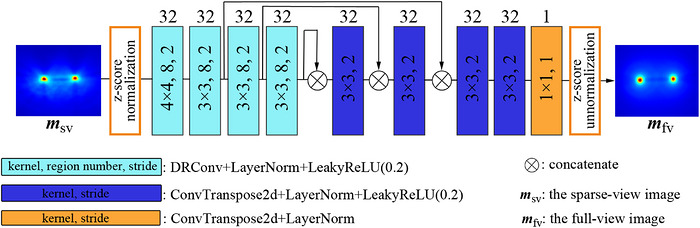
Structure of the post‐processing model^a^. (a) *z*‐score normalization: $m_{sv}^{\prime }=\frac{m_{sv}-\mu }{\sigma }$, *μ* and *σ* are the average and the standard deviation of *m*_sv_ respectively; *z*‐score unnormalization: the inverse calculation of *z*‐score normalization; DRConv, dynamic region‐aware convolution.^49^ The number on the top of the rectangle represents the filter number.

The post‐processing model's structure is exhibited in Figure 4. It uses a U‐net backbone to effectively fuse multi‐scale features.^50^ We replace Batch Normalization (BatchNorm) with Layer Normalization (LayerNorm)^48^ in all convolutional blocks. BatchNorm normalizes feature maps across the batch dimension for each feature channel using statistics computed over the mini‐batch. While this improves optimization stability, it introduces dependencies between samples and may be suboptimal for tasks where the desired mapping is inherently sample‐specific. In contrast, LayerNorm normalizes across feature dimensions within each individual sample, making it independent of batch size and sample composition. This property makes LayerNorm particularly suitable for our task of learning the mapping from sparse‐view to full‐view images, where the enhancement relies mainly on the intrinsic structure of each input rather than batch‐level statistics. Therefore, LayerNorm provides a more appropriate normalization strategy for our framework. To further improve the model's effectiveness in enhancing image, we proposed the DRConv^49^ to use different filters to correct image distortion according to its distortion patterns.

### 2.3 Experimental setup for electroacoustic signal acquisition

#### 2.3.1 Experiment overview

The experiments consist of two studies: (a) a linear‐array probe study using water‐tank data for training and both water‐tank and mouse data for testing, and (b) a convex‐array probe study with both training and testing conducted on water‐tank data. Study (b) extends Study (a) to evaluate EXPP‐Net under different probe geometries. The data acquisition settings, sample sizes, and data splitting strategy are summarized in Table 2. In the following subsections, we detailed the experiments.

**TABLE 2:** Summary of experimental settings.^a^

		Data acquisition settings									
			Electrical pulse						Data splitting strategy^d^		
Study type	Data source	Electric Field Strength^b^ (Volts/cm)	Width (ns)	Amplitude (kV)	Repetition frequency (Hz)	Probe sampling rate (MHz)	Electrode number	Sample size^c^	Training	Validation	Test
Linear‐array probe study	Water tank	1200∼2400	100	0.6∼1.2	20	40	2	54	34(2190)	10(600)	10(600)
	Mouse	4000	100	2.0	20	40	2	1	–	–	1(1)^e^
Convex‐array probe study	Water tank	2000∼8000	100	1.0∼4.0	50	40	2	22	18(2160)	2(240)	2(240)

^a^ Abbreviations: ‐, not applicable.

^b^ Electric Field Strength = Amplitude/ Distance between electrodes; In our study, distance between electrodes = 5 mm.

^c^ For water‐tank data, each sample is defined by a given electrode position and voltage, and comprises 60, 90 or 120 views. These views are obtained by rotating the electrode pair around a nominal pair center to collect signals at uniformly distributed angles over 360°. For mouse data, samples are defined in the same way but consist of only one single view.

^d^ Values are presented as the number of samples (number of views). No sample overlap exists among the training, validation, and test sets. The inputs to the deep learning model are counted based on the number of views.

^e^ Testing on mouse data is performed using a model trained on water‐tank data without fine‐tuning.

#### 2.3.2 Water‐tank experimental setup with a linear‐array probe

**FIGURE 5:**
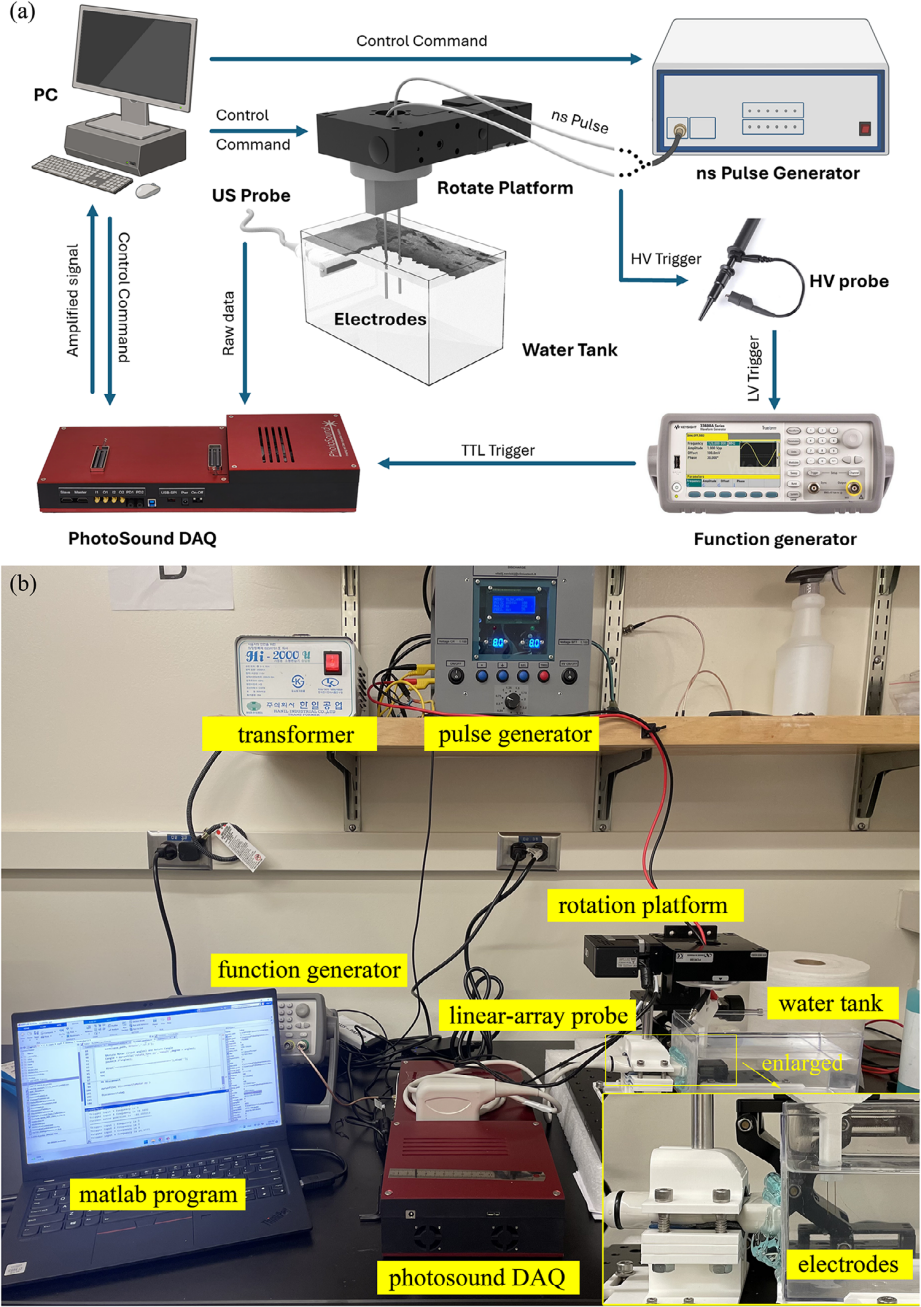
Experimental setup for the linear‐array probe study in a water tank: (a) schematic illustration and (b) photograph.

Figure 5 shows the experiment setup for data generation. Two tungsten electrodes (57400, A&M System, USA) were placed in a water tank. The two electrodes’ ends were fixed to a rotating platform (FCR100, Newport Corporation, USA). A custom nanosecond electrical pulse generator (VilniusTECH, Vilnius, Lithuania) was connected to the two electrodes and, via them, delivered electrical pulses to the objects. The electrical pulses were with a duration of ≤100 nanoseconds, an adjustable amplitude range of 0∼2 kV and a variable repetition frequency of 1 Hz∼1 MHz. Further details on the electrical excitation and probe–electrode geometry are provided in Supplementary Materials S4–S5 (Table S2 for electrical excitation; Figure S7 and Table S3 for probe–electrode geometry).

The ultrasound signal stimulated by the electrical pulses was detected by a clinical 128‐channel linear‐array ultrasound probe (L12‐5L60N, Telemed Medical Systems, Italy), and were in parallel digitized by a 128‐channel data acquisition (DAQ) system (Photosound, USA) with a sampling rate of 40 MHz and a depth of 12 bits. To capture such ultrasound signal, a high‐voltage probe (P4250, Keysight Technologies, USA) was used to detect the aforementioned high‐voltage pulses. The pulses were subsequently attenuated and transferred to a function generator (33600A, Keysight Technologies, USA) to output a standard TTL trigger signal to synchronize the data acquisition (see Supplementary Materials S6 for more ultrasound data acquisition and processing details).

The ultrasound probe was placed outside the tank to avoid the influence of electrical field on the probe. A hole was cut in the tank's side and covered by a polyethylene film for the coupling of the probe through the ultrasound gel. We used a Matlab program^2^ to control the entire system, including releasing the electrical pulses, rotating the electrodes and storing the ultrasound signals.

#### 2.3.3 In Vivo Experimental Setup with a Linear‐Array Probe

**FIGURE 6:**
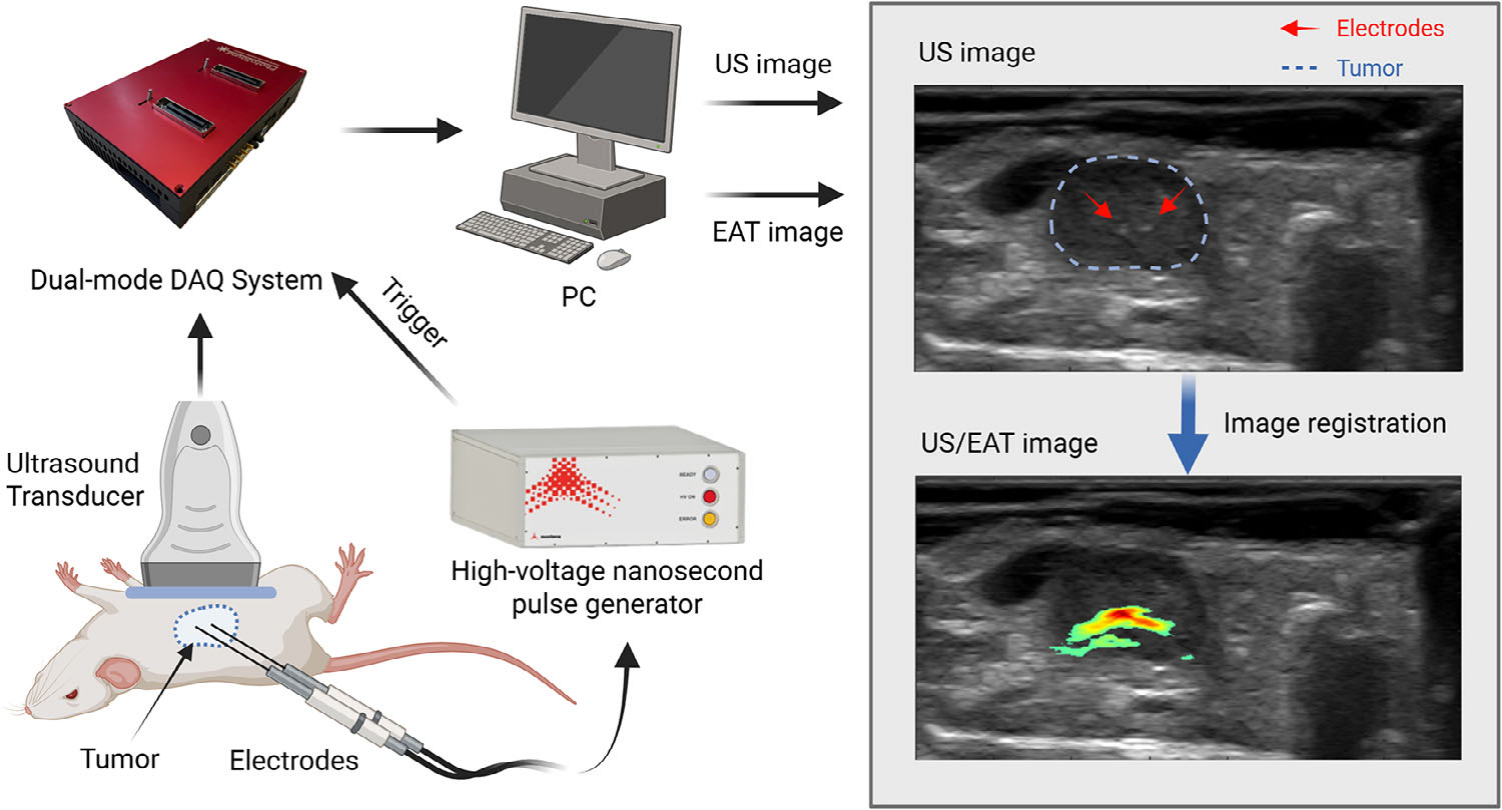
In vivo experimental setup with a linear‐array probe.

The animal experiment (n = 1 mouse), as shown in Figure 6, was conducted in accordance with the U.S. guidelines for the care and use of laboratory animals and was approved by the Institutional Animal Care and Use Committee (IACUC, protocol number AUP‐22‐043). Orthotopic prostate tumors were established by injecting Myc‐CaP murine prostate cancer cells into a NOD/SCID mouse. Tumor growth was monitored by measuring the tumor diameter using calipers. When tumors reached approximately 10 mm in size, IRE treatment was performed. Mouse was anesthetized intraperitoneally with ketamine (80–100 mg/kg) and xylazine (10–12.5 mg/kg). After confirming adequate anesthesia, abdominal hair was removed using depilatory cream. The mouse was placed supine on a heating pad, and a linear ultrasound probe (L12‐5N40‐A4, Telemed Ultrasound Medical System, Lithuania) was coupled to the abdominal skin using ultrasound gel. B‐mode ultrasound imaging was performed to localize the tumor. The tumor was exteriorized using forceps, and a custom‐made electrode (diameter: 514 µm, A&M Systems, USA) was inserted laterally into the tumor center under ultrasound guidance to avoid critical organs. A total of 150 pulses, each with a width of 100 ns, were delivered at 20 Hz and an amplitude of 2000 V. Ultrasound and EAT signals were acquired synchronously throughout the procedure. No adverse events were observed during the experiment. The dual‐modality imaging data were processed using MATLAB (2021b, MathWorks, USA) for signal processing, image reconstruction, and co‐registration.

#### 2.3.4 Water‐Tank Experimental Setup with a Convex‐Array Probe

For experiments based on convex array data acquisition, an updated nanosecond pulse generator (PGR‐10KV‐50R‐01‐V1, Montena, Switzerland) was employed, capable of delivering electrical pulses with pulse widths ranging from 60 to 160 ns at amplitudes up to 10 kV and repetition rates of up to 2000 Hz. A pair of clinical electrodes (20400103, AngioDynamics, USA) was modified to interface with this system for pulse delivery. The transrectal probe (BIPC8, Telemed Medical Systems, Italy) was positioned laterally to the electrodes in the water tank using a custom 3D‐printed holder, and a dedicated latex probe cover was used to electrically isolate the probe from external currents. A function generator (DG535, Stanford Research Systems, USA) was used to receive signals from the monitoring port of the pulse generator and to synchronize data acquisition. All other components and procedures were identical to those used in the linear array data acquisition system.

#### 2.3.5 Water‐Tank Data Generation

54 and 22 datasets were acquired in linear‐ and convex‐array probe studies respectively. Different datasets corresponded to various distances and angles from the two electrodes to the probe^3^, and were acquired under different voltages (1200∼8000 Volts/cm). For each dataset, we used the probe to collect signals from 60, 90 or 120 equally distributed views over 360°. To get a full‐view image as ground truth, all single‐view sinograms were reconstructed to their corresponding single‐view images using the back‐projection algorithm, and then were reconstructed into a full‐view image by rotating and averaging them as stated in Jiang et al.’s report^15^ (see Supplementary Materials S7 for more ground truth construction details).

### 2.4 Deep Learning Model Training and Evaluation

#### 2.4.1 Water‐Tank Data Splitting for Linear‐ and Convex‐Array Probe Studies

As shown in Figure 1, our EXPP‐Net's input variable includes a single‐view sinogram (***u*** *_α_*). Its intermediate outputs are four sinograms (***u*** *_α_*_‐60°_, ***u*** *_α_*_‐24°_, ***u*** *_α_*_+24°_ and ***u*** *_α_*_+60°_) and its final output is a full‐view image (***m***_fv_). A total of 3390 and 2640 view sets (i.e., {***u*** *_α_*, ***u*** *_α_*_+_ *_γ_* |*_γ_*_= ‐60°, ‐24°, 24°, 60°_, ***m***_fv_}) were collected for the linear‐ and convex‐array probe studies, respectively, and split into training/validation/test sets of 2190/600/600 and 2160/240/240 as summarized in Table 2.

#### 2.4.2 Model Training

The EXPP‐Net consists of two models, namely an extrapolation model and a post‐processing model. They were both updated using Adam^51^ due to its adaptive learning rate and stable convergence in non‐convex optimization as stated in Ian Goodfellow et al.^52^ The EXPP‐Net was first trained and evaluated on linear‐array probe water‐tank data for the linear‐array probe study. The resulting pretrained EXPP‐Net was then fine‐tuned via transfer learning^53^ using convex‐array probe water‐tank data and subsequently evaluated for the convex‐array probe study. Both studies adopted the training settings listed in Table 3. The methods to mitigate overfitting and underfitting are provided in Supplementary Materials S8, where loss curves over epochs (Figure S8) are included as supporting evidence.

**TABLE 3:** The training settings for different models.

	Adam^a^						
Models	learning rate^b^	*β*_1_	*β*_2_	*ɛ*	batch size	Epoch	optimal model
Extrapolation model	10^−3^	0.9	0.999	10^−8^	4	400	The one with a minimum loss on validation set
Post‐processing model	10^−3^	0.9	0.999	10^−8^	1	235	

^a^ The parameters in Adam were set as the default hyperparameters as recommended by Diederik P. Kingma et al.^51^.

^b^ The learning rate is reduced by a factor of 0.1, if no loss decrease on the training set is seen for 3 epochs, to improve training efficiency and convergence stability as suggested by Kaichao You et al.^54^.

In linear‐array probe study, the extrapolation model's loss function is a weighted mean square error (MSE):

(1) $$\begin{array}{cc} Loss_{extrapolation,linear} & =\frac{1}{N}\;\sum {\left(W\cdot \left(u-\hat{u}\right)\right)}^{2}\\ W & =4\times \frac{\hat{u}-min\left(\hat{u}\right)}{\max\left(\hat{u}\right)-min\left(\hat{u}\right)}+1 \end{array}$$

in which ***u*** and $\hat{u}$ are the estimated sinogram and its ground truth respectively. *N* represents the pixel number in $\hat{u}$. ***W*** is a weight matrix sharing the same size with $\hat{u}$. The max(·) and min(·) mean the maximum and minimum operators respectively.

In convex‐array probe study, because of the large signal range variance (spanning approximately three orders of magnitude), Equation (1) was heavily dominated by high‐magnitude samples, resulting in insufficient optimization for relatively‐low‐magnitude samples. Therefore, the relative root mean square error (rRMSE) was adopted as the loss function for the extrapolation model:

(2) $$Loss_{extrapolation,convex}=\sqrt{\frac{1}{N}\sum {\left(\frac{u-\hat{u}}{\max\left(\hat{u}\right)}\right)}^{2}}$$

where all symbols in Equation (2) are defined as in Equation (1). A detailed rationale for adopting different loss functions is provided in Supplementary Material S2 (3).

The post‐processing model's loss function is a SSIM derivative:

(3) $$\begin{array}{cc} & \mathrm{Los}s_{\mathrm{post}-\mathrm{proc}\mathrm{essi}\mathrm{ng}}=1-\mathrm{SSIM}\left(m_{\mathrm{fv}},{\hat{m}}_{\mathrm{fv}}\right)\\ & \mathrm{SSIM}\left(x,y\right)=\frac{\left(2\mu_{x}\mu_{y}+c_{1}\right)\left(2\sigma_{\mathrm{xy}}+c_{2}\right)}{\left(\mu_{x}^{2}+\mu_{y}^{2}+c_{1}\right)\left(\sigma_{x}^{2}+\sigma_{y}^{2}+c_{2}\right)}{|}_{c_{1}={\left(0.01\times L\right)}^{2},c_{2}={\left(0.03\times L\right)}^{2}} \end{array}$$

wherein ***m***_fv_ is the estimated full‐view image. Its ground truth is ${\hat{m}}_{fv}$. *μ_x_* and *μ_y_* are the averages of *x* and *y* respectively. $\sigma_{x}^{2}$ and $\sigma_{y}^{2}$ are the variances of *x* and *y* respectively. *σ_xy_* is the covariance of *x* and *y*. *L* represents the dynamic range of the pixel values in ${\hat{m}}_{fv}$.

#### 2.4.3 Evaluation metrics

The EXPP‐Net performance was evaluated using RMSE, SSIM and the iso‐pressure line DICE coefficients, following the same metrics as the benchmark method.^15^

(4) $$RMSE=\sqrt{\frac{1}{N}\sum {\left(m_{fv}-{\hat{m}}_{fv}\right)}^{2}}$$

(5) $$\begin{array}{c} DICE\left(r\right)=\frac{2XY}{X+Y}\;{|}_{r=10\%,20\%,30\%,40\%,50\%,60\%,70\%}\\ X=\left(x_{ij}\right),x_{ij}=\left\{\begin{array}{c} 0,if\;m_{ij}<r\cdot max\left({\hat{m}}_{fv}\right)\;and\;m_{ij}\in m_{fv}\\ 1,if\;m_{ij}\geq r\cdot max\left({\hat{m}}_{fv}\right)\;and\;m_{ij}\in m_{fv} \end{array}\right.\\ Y=\left(y_{ij}\right),y_{ij}=\left\{\begin{array}{c} 0,if\;{\hat{m}}_{ij}<r\cdot max\left({\hat{m}}_{fv}\right)\;and\;{\hat{m}}_{ij}\in {\hat{m}}_{fv}\\ 1,if\;{\hat{m}}_{ij}\geq r\cdot max\left({\hat{m}}_{fv}\right)\;and\;{\hat{m}}_{ij}\in {\hat{m}}_{fv} \end{array}\right. \end{array}$$

in which ***m***_fv_ is the estimated full‐view image. Its ground truth is ${\hat{m}}_{fv}$. *N* refers to the pixel number in ${\hat{m}}_{fv}$. The max(·) is the maximum operator. X is a binary matrix with the same size of ***m***_fv_. Y is a binary matrix sharing the same size of ${\hat{m}}_{fv}$.

All metrics were computed over the entire image, including background pixels without special processing. Note that the background pixels with lower values may slightly dilute the contribution of the foreground region to the overall RMSE and SSIM values. However, DICE is largely insensitive to the background. DICE is calculated based on the overlap of the foreground mask. Therefore, the evaluation metrics still accurately reflect the performance of the model on the region of interest.

#### 2.4.4 Comparison with Other Works

Our EXPP‐Net was compared with one published model^15^ which also enhanced the EAT quality from a single‐view image. The comparative model was trained using the same linear‐array probe water‐tank data and the same training strategy as our work. A technical comparison between this benchmark and our EXPP‐Net is provided in Supplementary Material S9, along with the rationale for selecting this benchmark as a representative standard in this field (Figure S9 illustrates the single‐view artifact that is the primary target of both this benchmark and our work).

#### 2.4.5 Ablation study

In our work, we proposed (a) the self‐designed convolutions (i.e., the RotDRConv and RotTransDRConv) in the extrapolation model to extrapolate the sinograms at other angles, and (b) the DRConv in the post‐processing model to correct image distortion. To further study their performances, we replaced them with the conventional convolution (abbreviated as Conv), and compared the results with our EXPP‐Net. The detailed ablation settings are listed in Table 4.

**TABLE 4:** Ablation study settings.^a^

Model	Ablation settings
EXPP‐Net	Extrapolation model(RotDRConv, RotTransDRConv)+Post‐processing model(DRConv)
model 1	Extrapolation model(RotDRConv, RotTransDRConv)+Post‐processing model(Conv)
model 2	Extrapolation model(Conv)+Post‐processing model(DRConv)
model 3	Extrapolation model(Conv)+Post‐processing model(Conv)

^a^ Abbreviation: (·): the convolution abbreviation in the parenthesis represents the one used in the model. The RotDRConv and RotTransDRConv share the same meanings in Figure 3(b); DRConv, dynamic region‐aware convolution.

#### 2.4.6 Statistical Methods

We conducted statistical analyses to evaluate the proposed EXPP‐Net against (1) the ground truth, (2) a benchmark method, and multiple EXPP‐Net variants.

To account for repeated measurements and within‐sample angular variability, we employed a linear mixed‐effects model (LMM). In this framework, model was treated as a fixed effect, while sample was included as a random intercept to account for between‐sample variability.

To explicitly model systematic variations across rotation angles, angular dependence was incorporated using sine and cosine basis functions, capturing the intrinsic periodicity of the 360° rotation (i.e., a first‐order Fourier series expansion). This formulation enables the model to disentangle method‐related effects from both between‐sample variability and within‐sample angular structure, thereby improving statistical efficiency and reducing bias in performance estimation.

1. Comparison against ground truth

To estimate the uncertainty of each performance metric while accounting for within‐sample correlations, we adopted a cluster bootstrap procedure. Specifically, bootstrap resampling was performed at the sample level with replacement, preserving all observations within each resampled sample.

For each bootstrap replicate, the LMM was refitted to the resampled dataset, including angular terms (sine and cosine) and a random intercept for sample. Model performance was evaluated by comparing the LMM‐fitted values with the observed metric within each resampled dataset. This procedure was repeated 1,000 times, and the 95% confidence intervals (CIs) were obtained from the 2.5th and 97.5th percentiles of the resulting bootstrap distribution.

2. Comparison against benchmark and EXPP‐Net variants

Statistical significance of fixed effects was assessed using *p*‐values from Wald tests based on the estimated coefficients of the LMM. Multiple comparisons were controlled using the Benjamini–Hochberg procedure, and a significance level of *p* < 0.05 was adopted.

Effect sizes were computed as standardized fixed‐effect coefficients (*β*/*σ*)^55^, ^56^, ^57^ with *β* as the fixed‐effect estimate and *σ* as the residual standard deviation of the model. Effect magnitudes (|*β*/*σ*|) were interpreted using Cohen's conventional benchmarks (0.2, 0.5, 0.8 for small, medium, and large effects, respectively)^55^. The corresponding 95% CIs were computed using the *t*‐distribution based on the estimated standard errors and associated degrees of freedom.

## 3 RESULTS

### 3.1 Quantitative results on linear‐array probe water‐tank data

**FIGURE 7:**
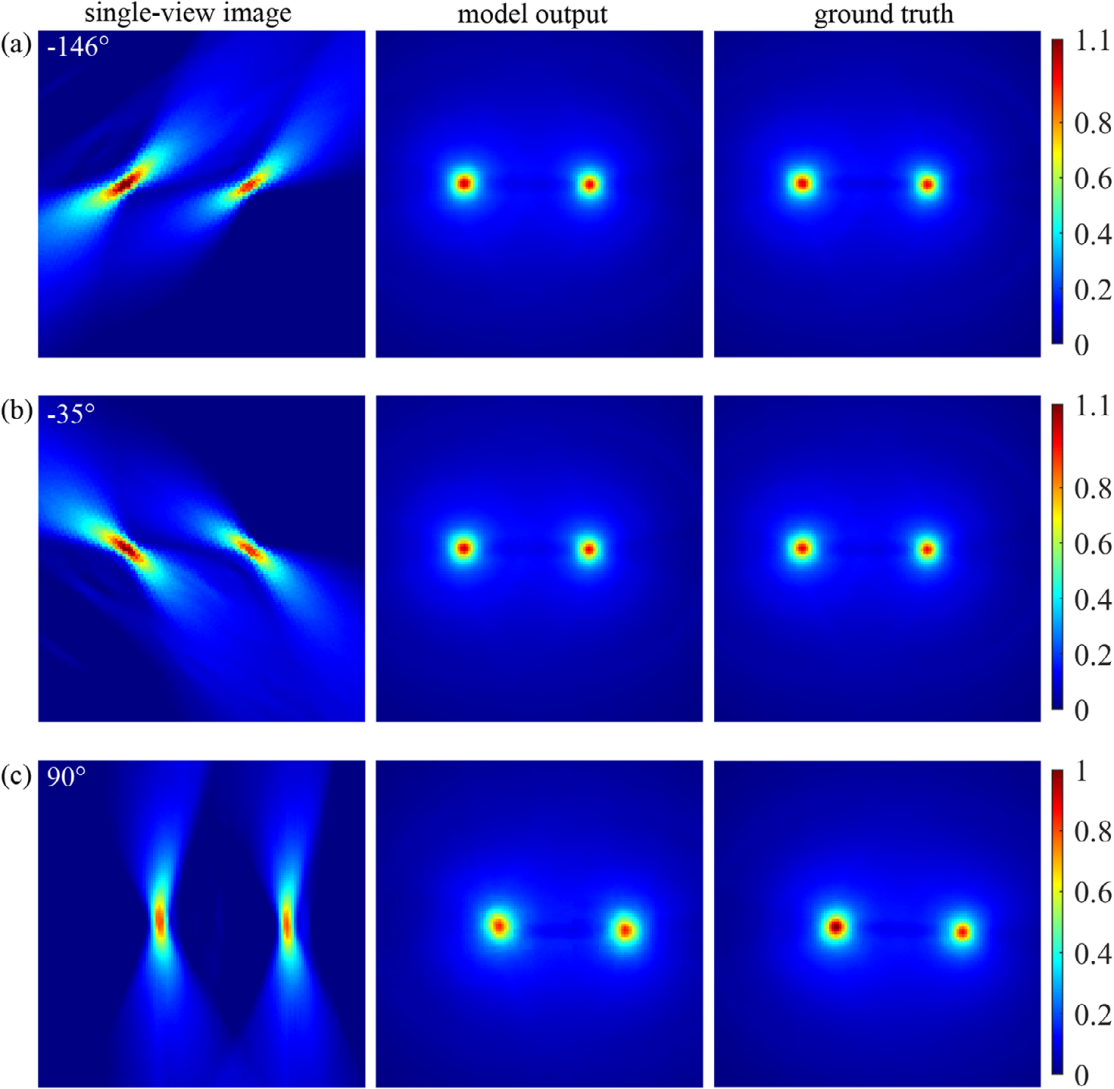
Three examples (a∼c) of using a linear‐array probe on water tank data for qualitative evaluation. All images are normalized according to their ground truths.

Table 5 summarizes the EXPP‐Net's quantitative results. The results show a high intensity agreement between the model output and ground truth, since the normalized RMSE is 0.0033 ± 0.0023 and SSIM is 0.9968 ± 0.0059. The structural shape was evaluated using the iso‐pressure line DICEs, and all DICEs present a median of ≥0.9450. Figure 7 shows several examples for qualitative visualization. The first column exhibits the single‐view images, which is obvious that the various angles present different spatial distortions. Despite that, our model outputs show substantial concordance with the ground truth, which demonstrates our model's effectiveness in correcting single‐view distortions.

**TABLE 5:** EXPP‐Net Quantitative Results on Linear‐Array Probe Water‐Tank Data.^a^

	View‐level statistics					
Metrics	Average ± std	median	70^th^ percentile	90^th^ percentile	95^th^ percentile	Sample‐level 95% CI
**RMSE**↓	0.0033 ± 0.0023	0.0023	0.0031	0.0059	0.0087	[0.0022, 0.0041]
**SSIM**↑	0.9968 ± 0.0059	0.9990	0.9993	0.9996	0.9996	[0.9949, 0.9989]
**DICE(10%)**↑	0.9749 ± 0.0250	0.9837	0.9881	0.9920	0.9931	[0.9675, 0.9832]
**DICE(20%)**↑	0.9565 ± 0.0352	0.9708	0.9768	0.9810	0.9832	[0.9448, 0.9696]
**DICE(30%)**↑	0.9516 ± 0.0370	0.9675	0.9737	0.9799	0.9856	[0.9357, 0.9690]
**DICE(40%)**↑	0.9517 ± 0.0464	0.9721	0.9795	0.9855	0.9888	[0.9323, 0.9734]
**DICE(50%)**↑	0.9406 ± 0.0634	0.9692	0.9776	0.9845	0.9880	[0.9166, 0.9711]
**DICE(60%)**↑	0.9260 ± 0.0877	0.9636	0.9730	0.9912	1.0000	[0.8873, 0.9669]
**DICE(70%)**↑	0.8862 ± 0.1439	0.9450	0.9587	0.9773	0.9863	[0.8344, 0.9511]

^a^ Abbreviation: ↑, higher is better; ↓, lower is better; 95% CI, 95% confidence interval. The images are normalized to [0, 1] to calculate these metrics; std, standard deviation.

### 3.2 Qualitative Results on In Vivo Linear‐Array Probe Data

**FIGURE 8:**
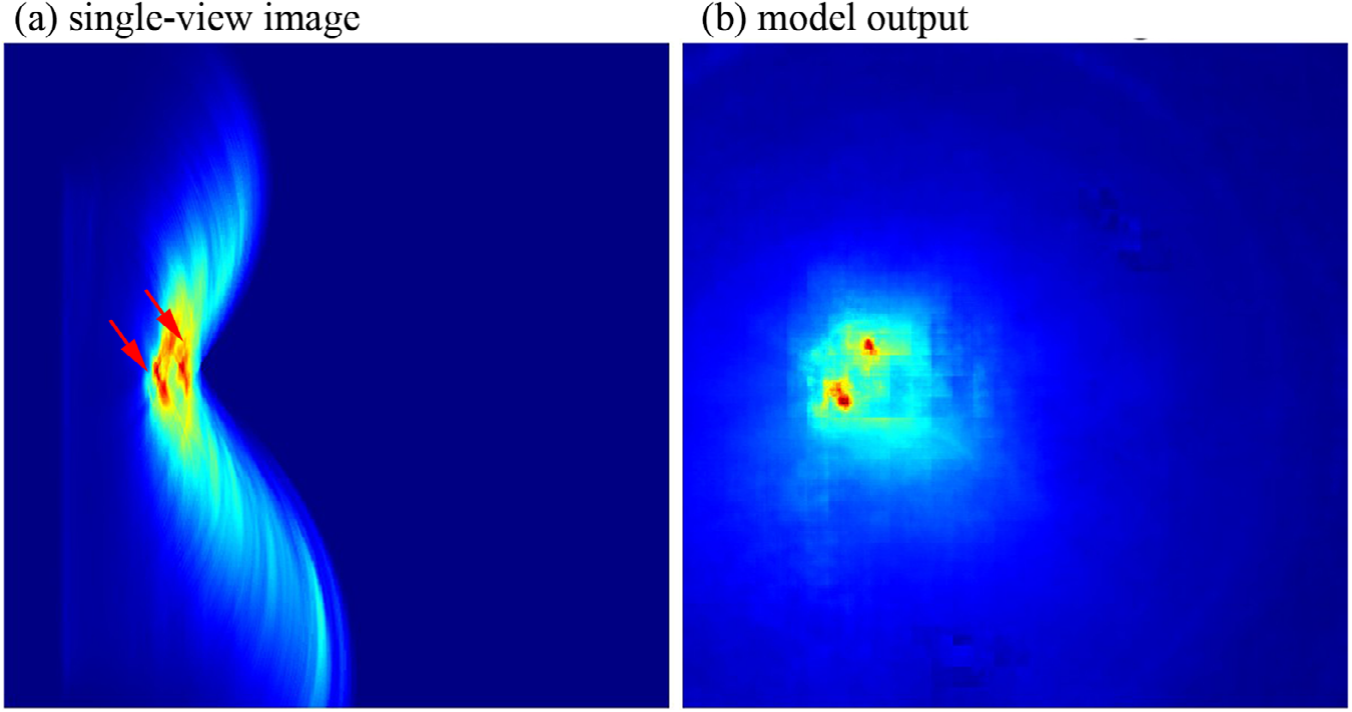
EXPP‐Net Result on In Vivo Linear‐Array Probe Data. In (a), the two red arrows denote the two electrodes.

Figure 8 shows the model output on mouse data. By comparing Figure 8(a) and (b), we can find that our model corrects the image distortions effectively, and restores the two hot spots at the two electrodes’ positions and their surrounding signals. The quantitative evaluation on the mouse result wasn't performed, since the full‐view ground truth can't be acquired by rotating electrodes or probe. In our future work, to compensate the translation error from water tank to in‐vivo data, we will collect more animal data to fine tune our model weights.

### 3.3 Quantitative Results on Convex‐Array Probe Water‐Tank Data (Extended Study)

**FIGURE 9:**
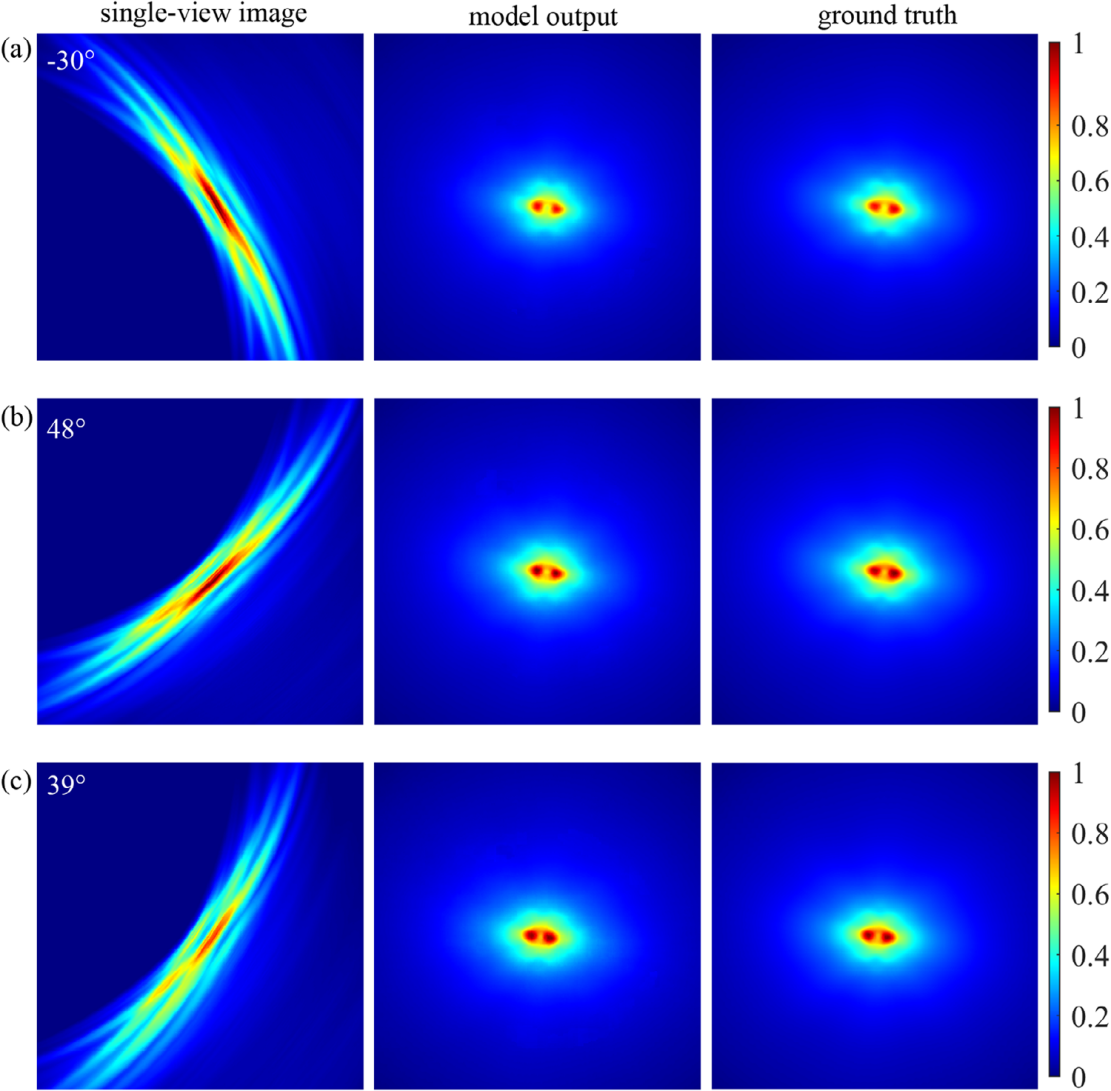
Three examples (a∼c) of EXPP‐net results on convex‐array probe water‐tank data for qualitative evaluation. All images are normalized according to their ground truths.

Table 6 exhibits the quantitative results. Although with a small‐size training dataset, our EXPP‐Net shows a good match with the ground truth, since the normalized RMSE is 0.0083 ± 0.0034 and SSIM is 0.9958 ± 0.0038. The iso‐pressure line DICEs with a median of ≥ 0.8837 demonstrate the accuracy on structural shape. Figure 9 illustrates three examples for qualitative evaluation. Although the single‐view images in first column show different distortion patterns with the ones in Figure 7, our model reaches a good match with the ground truth after transfer learning using small‐quantity data.

**TABLE 6:** EXPP‐Net Quantitative Results on Convex‐Array Probe Water‐Tank Data.^a^

	View‐level statistics					
Metrics	Average ± std	median	70^th^ percentile	90^th^ percentile	95^th^ percentile	Sample‐level 95% CI
**RMSE**↓	0.0083 ± 0.0034	0.0075	0.0096	0.0125	0.0154	[0.0081, 0.0085]
**SSIM**↑	0.9958 ± 0.0038	0.9970	0.9981	0.9988	0.9990	[0.9955, 0.9961]
**DICE(10%)**↑	0.9611 ± 0.0179	0.9650	0.9718	0.9790	0.9819	[0.9603, 0.9620]
**DICE(20%)**↑	0.9537 ± 0.0244	0.9585	0.9686	0.9776	0.9846	[0.9484, 0.9591]
**DICE(30%)**↑	0.9498 ± 0.0252	0.9554	0.9678	0.9776	0.9807	[0.9492, 0.9505]
**DICE(40%)**↑	0.9309 ± 0.0357	0.9411	0.9555	0.9687	0.9715	[0.9308, 0.9310]
**DICE(50%)**↑	0.9240 ± 0.0373	0.9311	0.9482	0.9652	0.9686	[0.9174, 0.9307]
**DICE(60%)**↑	0.9222 ± 0.0476	0.9347	0.9511	0.9686	0.9712	[0.9113, 0.9330]
**DICE(70%)**↑	0.8581 ± 0.1052	0.8837	0.9148	0.9446	0.9546	[0.8398, 0.8763]

^a^ Abbreviation: ↑, higher is better; ↓, lower is better; 95% CI, 95% confidence interval. The images are normalized to [0, 1] to calculate these metrics; std, standard deviation.

### 3.4 Comparison Results with Other Works

**FIGURE 10:**
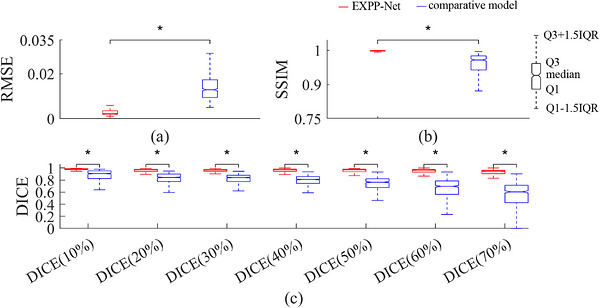
View‐level comparison results of our EXPP‐Net and comparative model^a^. ^a^Q1: 25^th^ percentile; Q3: 75^th^ percentile; IQR = Q3‐Q1; *, statistical significance (*p* < 0.05; linear mixed‐effects model; sample‐level random effects; not view‐level inference). A better performance corresponds to a lower RMSE, a higher SSIM and a higher DICE. These metrics are calculated by normalizing the images according to their ground truths.

The view‐level comparison results are exhibited in Figure 10, which illustrates the distribution of model outputs across different rotation angles (views). The sample‐level statistical analysis is reported in Table 7 to ensure valid statistical inference at the independent sample level. The view‐level boxplots in Figure 10 demonstrate that our EXPP‐Net achieves better performance, with lower RMSE and higher SSIM. All of the iso‐pressure line DICEs approximate the best (i.e., DICE = 1) compared with the comparative model. The statistical results in Table 7 further support these findings, with all comparisons showing *p *< 0.05 and the magnitude of effect size |*β*/*σ*| > 0.8.

**TABLE 7:** Sample‐level statistical comparison of EXPP‐Net and comparative model across multiple evaluation metrics.^a^

Metric	*p*‐value^b^	Standardized effect size (*β*/*σ*)^c^, 95% CI
**RMSE** ↓	<1.00 × 10^−300*^	3.23, [3.12, 3.35]
**SSIM** ↑	2.10 × 10^−194*^	−2.09, [−2.20, −1.98]
**DICE(10%)**↑	1.02 × 10^−160*^	−1.83, [−1.94, −1.72]
**DICE(20%)**↑	1.57 × 10^−218*^	−2.28, [−2.39, −2.16]
**DICE(30%**↑	7.09 × 10^−309*^	−3.00, [−3.12, −2.89]
**DICE(40%)**↑	7.05 × 10^−301*^	−2.94, [−3.05, −2.82]
**DICE(50%)**↑	1.26 × 10^−267*^	−2.67, [−2.78, −2.55]
**DICE(60%)**↑	9.64 × 10^−241*^	−2.45, [−2.56, −2.34]
**DICE(70%)**↑	1.55 × 10^−224*^	−2.32, [−2.44, −2.21]

^a^ Abbreviations: ↑, higher is better; ↓, lower is better; 95% CI, 95% confidence interval.

^b^ Statistical significance was defined as *p*‐value < 0.05 and indicated by *.

^c^ The magnitude of standardized effect size (*β*/*σ*) was interpreted using conventional benchmarks (|*β*/*σ*| = 0.2, 0.5, 0.8 for small, medium, and large effects, respectively), noting that these thresholds are approximate. The sign indicates the direction of the effect. Here, *β* = Metric(comparative model) − Metric(EXPP‐Net). Thus, *β* > 0 favors EXPP‐Net for lower‐is‐better metrics, whereas *β* < 0 favors EXPP‐Net for higher‐is‐better metrics.

### 3.5 Ablation study results

**FIGURE 11:**
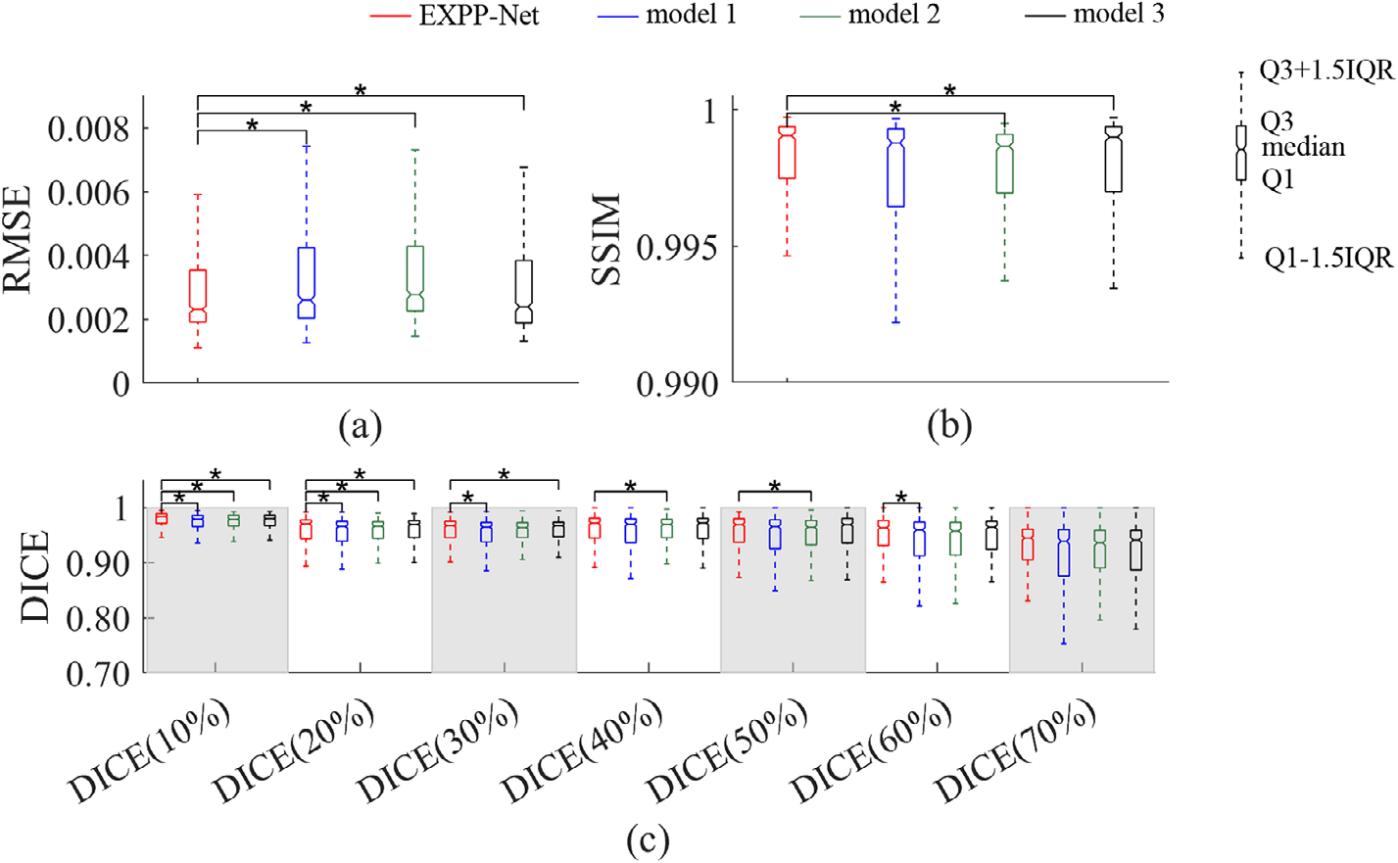
View‐level ablation study results^a^. ^a^Q1, 25^th^ percentile; Q3, 75^th^ percentile; IQR = Q3‐Q1; *, statistical significance (*p* < 0.05; linear mixed‐effects model; sample‐level random effects; not view‐level inference). The model 1∼3's ablation settings are listed in Table 4. A better performance corresponds to a lower RMSE, a higher SSIM and a higher DICE. These metrics are calculated by normalizing the images according to the ground truth's minimum and maximum values.

The view‐level boxplots are shown in Figure 11, and the sample‐level statistical analysis is reported in Table 8. Figure 11 demonstrates that our proposed EXPP‐Net reaches the best performance with statistically significant improvement (*p *< 0.05 in Table 8) in terms of RMSE, DICE(10%) and DICE(20%). Although the effect size is relatively small (|*β*/*σ*| ranging from 0.14 to 0.37 in Table 8), and statistical significance is not consistently observed across all remaining metrics and comparisons, the results exhibit a consistent performance trend in favor of EXPP‐Net. This is expected, since the compared models are closely related variants of EXPP‐Net, resulting in inherently smaller performance differences.

**TABLE 8:** Sample‐level statistical analysis for ablation study across multiple evaluation metrics.^a^

Metric		*p*‐value^b^	Standardized effect size (*β*/*σ*)^c^, 95% CI
RMSE↓	**EXPP‐Net vs. Model 1**	2.81 × 10^−4*^	0.23, [0.11, 0.34]
	**EXPP‐Net vs. Model 2**	1.01 × 10^−9*^	0.37, [0.26, 0.49]
	**EXPP‐Net vs. Model 3**	1.01 × 10^−4*^	0.25, [0.13, 0.36]
SSIM↑	**EXPP‐Net vs. Model 1**	8.76 × 10^−2^	−0.11, [−0.22, 0.01]
	**EXPP‐Net vs. Model 2**	2.69 × 10^−2*^	−0.14, [−0.25, −0.02]
	**EXPP‐Net vs. Model 3**	1.91 × 10^−4*^	−0.23, [−0.34, −0.12]
DICE(10%)↑	**EXPP‐Net vs. Model 1**	1.47 × 10^−5*^	−0.27, [−0.38, −0.16]
	**EXPP‐Net vs. Model 2**	4.90 × 10^−4*^	−0.22, [−0.34, −0.11]
	**EXPP‐Net vs. Model 3**	1.23 × 10^−5*^	−0.28, [−0.39, −0.17]
DICE(20%)↑	**EXPP‐Net vs. Model 1**	5.82 × 10^−6*^	−0.29, [−0.40, −0.17]
	**EXPP‐Net vs. Model 2**	2.69 × 10^−2*^	−0.14, [−0.25, −0.02]
	**EXPP‐Net vs. Model 3**	2.70 × 10^−4*^	−0.22, [−0.34, −0.11]
DICE(30%)↑	**EXPP‐Net vs. Model 1**	7.50 × 10^−3*^	−0.17, [−0.28, −0.06]
	**EXPP‐Net vs. Model 2**	5.37 × 10^−2^	−0.12, [−0.23, −0.00]
	**EXPP‐Net vs. Model 3**	7.12 × 10^−3*^	−0.17, [−0.28, −0.05]
DICE(40%)↑	**EXPP‐Net vs. Model 1**	1.62 × 10^−1^	−0.08, [−0.19, 0.03]
	**EXPP‐Net vs. Model 2**	9.80 × 10^−3*^	−0.17, [−0.28, −0.06]
	**EXPP‐Net vs. Model 3**	5.45 × 10^−2^	−0.12, [−0.23, −0.01]
DICE(50%)↑	**EXPP‐Net vs. Model 1**	1.62 × 10^−1^	−0.08, [−0.20, 0.03]
	**EXPP‐Net vs. Model 2**	1.16 × 10^−2*^	−0.16, [−0.27, −0.05]
	**EXPP‐Net vs. Model 3**	6.49 × 10^−1^	−0.04, [−0.15, 0.07]
DICE(60%)↑	**EXPP‐Net vs. Model 1**	3.82 × 10^−2*^	−0.13, [−0.25, −0.02]
	**EXPP‐Net vs. Model 2**	6.65 × 10^−2^	−0.11, [−0.22, 0.00]
	**EXPP‐Net vs. Model 3**	6.90 × 10^−1^	−0.03, [−0.14, 0.08]
DICE(70%)↑	**EXPP‐Net vs. Model 1**	8.76 × 10^−2^	−0.11, [−0.22, 0.00]
	**EXPP‐Net vs. Model 2**	8.01 × 10^−1^	0.01, [−0.10, 0.13]
	**EXPP‐Net vs. Model 3**	8.43 × 10^−1^	0.01, [−0.10, 0.12]

^a^ Abbreviations: ↑, higher is better; ↓, lower is better; 95% CI, 95% confidence interval.

^b^ Statistical significance was defined as *p*‐value < 0.05 and indicated by *.

^c^ The magnitude of standardized effect size (*β*/*σ*) was interpreted using conventional benchmarks (|*β*/*σ*| = 0.2, 0.5, 0.8 for small, medium, and large effects, respectively), noting that these thresholds are approximate. The sign indicates the direction of the effect. Here, *β* = Metric(Model) − Metric(EXPP‐Net). Thus, *β* > 0 favors EXPP‐Net for lower‐is‐better metrics, whereas *β* < 0 favors EXPP‐Net for higher‐is‐better metrics.

## 4 DISCUSSION

EAT is an emerging technique for guiding electrical‐based therapy and may be the only viable approach. Its potential was first demonstrated and reported by Xu et al.,^13^ using a ring‐array probe, in 2023. Due to the challenges of using a ring‐array probe in clinical practice, our work explores the feasibility of EAT imaging using a linear‐ and a convex‐array probes. Specifically, we study a deep learning model to tackle the image distortion problem caused by single‐view acquisition, since a linear or convex array probe can only capture signal at one angle to achieve real‐time monitoring.

In clinical applications, our models need to generate high‐quality images from single‐view EAT images to meet the demands in the following aspects: (a) verifying the signal strength in the high‐intensity region for quantifying the harm on cell; (b) verifying the treatment field structural shape from high‐intensity to low‐intensity regions, namely the signal falloff, for quantifying the cell damage regions; (c) achieving high performance in verification regardless of the angle of the single‐view acquisition.

For the above (a), our model shows a high quantitative performance for different signal intensities in the test cases, as shown in Table 5. For most cases, SSIM is > 0.99 (as indicated by the median SSIM = 0.9990 in Table 5) and RMSE is < 0.009 (as indicated by the 95^th^ percentile of RMSE = 0.0087 in Table 5). For (b), we used the iso‐pressure line DICEs to evaluate the signal structural shape and intensity fall‐off. As shown in Table 5, all DICEs show an average of ≥ 0.8862 and a median of ≥ 0.9450. For (c), the test cases include diverse distortion patterns caused by various single views. Our model exhibits a high generalization on correcting them, as visualized in Figure 7 and quantified in Table 5. The model output on mouse data, in Figure 8, further demonstrates our model's effectiveness. In contrast to the comparative model that enhances EAT image directly from single view reconstruction, we use the extrapolation model to extrapolate other 4 angle views and thus convert the single‐view distortion to 5‐angle sparse‐view distortion to alleviate the distortion before correction. As a result, the distortion pattern becomes relatively consistent, making it more feasible to correct the distortion using a fixed‐weight post‐processing model. Results in Figure 10 demonstrated the substantial improvement of the image quality using our method, compared to the previous method. The statistical results reported in Table 7 further support it, as all metrics achieved *p* < 0.05 and all effect sizes |*β*/*σ*| > 0.8.

To study our EXPP‐Net's performance on other ultrasound array geometries, we used transfer learning to optimize it on a small‐size dataset. Table 6 demonstrated its excellent performance, since RMSE reaches 0.0083 ± 0.0034 and SSIM is 0.9958 ± 0.0038, and all iso‐pressure line DICEs reached a median of > 0.8837.

### 4.1 Limitation and future direction

EAT is a relatively new treatment modality that's still under investigation. Our pilot experimental study demonstrated the potential of using deep learning to substantially improve the quality of single‐view EAT images. One limitation of the study is that the validation of the model has been carried out primarily in a homogeneous medium with a preliminary test in an animal scan. This is because EAT imaging is still at an early stage of investigation, and there have been limited animal studies and no patient studies for this novel modality. Our study represents one of the first studies to demonstrate the potential of generating high‐quality images from single‐view EAT acquisition. In the future, more comprehensive animal and patient studies are warranted to further evaluate the techniques in different settings.

Our proposed RotDRConv and RotTransDRConv are designed to fit the rotation‐induced signal variations. In addition to the application in EAT imaging, they, by extrapolating the data corresponding to other angles, are promising to tackle the undersampling problem caused by limited view acquisition in other imaging modalities, such as CT, cone‐beam CT or proton‐acoustic imaging, to improve the image quality with shortened scanning time or reduced imaging dose.

## 5 CONCLUSION

We have developed a novel EXPP‐Net deep learning model to substantially enhance the quality of single‐view EAT images to be comparable to full‐view EAT images under the evaluated settings, as demonstrated in the pilot experimental studies in phantom and animals. These results suggest that the technique has the potential to improve imaging performance for electroporation treatment evaluation, although further validation is required for real‐time clinical application. Future work will focus on further validating our self‐designed RotDRConv and RotTransDRConv under more diverse imaging conditions.

## CONFLICT OF INTEREST STATEMENT

All authors declare that they have no known conflicts of interest in terms of competing financial interests or personal relationships that could have an influence or are relevant to the work reported in this paper.

## Supporting information



Supporting Information

## Footnotes

1 This paper includes both linear‐array and convex‐array probe studies. Note that this figure illustrates the architecture used in the linear‐array probe study, whereas a slightly different architecture was adopted for the convex‐array probe study. More details are provided in Supplementary Materials S2 (Figures S2 and S3).

2 The script is available upon reasonable request.

3 The stage rotated the electrodes over 360°, and then adjusted the distance between the electrodes and the probe.
