# Supplementary material for: A single‐view‐based electroacoustic tomography imaging using deep learning for electroporation monitoring
Source: Med Phys. 2026 Aug 10;53(8):e70623. doi: 10.1002/mp.70623 (PMC13455628; doi:10.1002/mp.70623)
Supplement: Supplementary file 1 [file MP-53-0-s001.zip › mp70623-sup-0010-SuppMat.doc]

**S1. Technical Challenges of Existing Approaches and Methodological Adjustments in EXPP-Net**

Table S1 summarizes the technical challenges of existing approaches and the corresponding methodological adjustments in our proposed EXPP-Net. Fig. S1 illustrates the advantages of EXPP-Net by integrating both extrapolation and image enhancement within the imaging field. Specifically, the contributions of EXPP-Net are twofold: (a) a proposed extrapolation technique that explicitly aligns with underlying data regularities, enabling more accurate estimation of missing data; and (b) an image enhancement approach that leverages (a) to transform single-view distortion into a five-view setting with a more uniform distortion pattern, thereby simplifying the task compared to existing methods and improving performance.

**Table S1. Technical Challenges and Corresponding Methodological Adjustments**

| **Existing methods** | | | | **Our methodological adjustments** |
| --- | --- | --- | --- | --- |
| **References^a^** | **Method Category** | **Technical Challenges** | **Technical Challenge Notes** |  |
| [33]^b^ | Extrapolation | Limited to a specific scanning configuration | The limited angle range is not an explicit input to the network, which implicitly constrains the model to a specific limited-angle setting (e.g., 0°–15°) and may limit its generalization to other scanning configurations (e.g., 90°–120°) without re-training, transfer learning, or fine-tuning with new data. | The extrapolation angle is provided as an input to the network^c^, allowing it to adaptively adjust its weights for improved sinogram extrapolation. |
| [38-40] | Inpainting using generative adversarial networks (GAN) | (a) Limited by the difficulty of obtaining training samples that cover all possible variations.  (b) The task is closer to interpolation, where missing data lies within the observed data range | (a) GAN-based models learn the probability distribution of the training data and generate outputs that conform to this distribution. As a result, their performance may degrade when the test data differs from the training data. | Not suitable for our work. The reasons are:  (a) While anatomical variations in CT imaging are relatively small—note that the referenced works are all developed for CT imaging—our task involves substantial variations in treatment parameters (e.g., voltage and pulse duration in electroporation), making it difficult to obtain sufficiently diverse training samples.  (b) In contrast, our task is based on a single observation, where all missing data lies outside the available data range, making it an extrapolation problem rather than an inpainting task. |
| [15, 43] | Direct enhancement | Limited to a single distortion pattern. | Without preprocessing to enforce uniform distortion patterns, a fixed-parameter model can only offer a suboptimal correction across different data and cannot achieve high precision. (as shown in the Direct Enhancement panel in Fig. S1) | Before enhancement, we apply extrapolation to generate four additional sinograms, converting the task from single-view distortion correction to five-view distortion correction with a more uniform pattern that is easier to learn. (as shown in the Preconditioned Enhancement panel in Fig. S1) |

^a^The reference numbers in this table are aligned with those in the main manuscript, rather than the supplementary materials.

^b^While the work includes additional modules, the authors consider the sinogram extrapolation module as the main contribution, so we categorize it under extrapolation.

^c^In addition to angle-modulated weights, our extrapolation model incorporates sub-region-modulated weights to better capture data regularities in rotational acquisition, which have not been explored in previous works. More details are provided in Supplementary Materials S3.

**Fig. S1. Comparison between Direct and Preconditioned Enhancements.^a^**

^a^ Notations: *f_θ_*_1_, *f_θ_*_2_: enhancement models with parameters *θ*_1_ and *θ*_2_ for 100% correction of the images acquired at 0° and 92°, respectively; *θ*: parameters of the enhancement model learned via deep learning.

**S2. Details of Extrapolation model for linear- and convex-array probe studies**

The overall architecture of the proposed extrapolation model is shown in Fig. 3. In this section, we provide further details of the network implementation. Note that the extrapolation models used for the linear- and convex-array probe studies differ slightly to accommodate their respective data distributions. In the following subsections, we (1) explicitly describe these differences, (2) discuss their underlying reasons, and (3) analyze the differences in loss functions used in the two studies for training extrapolation models.

**(1) Detailed Model Structures for Linear- and Convex-array Probe Studies**

Fig. S2 exhibits the details of extrapolation models for linear- and convex-array probe studies. As highlighted by the red arrows in Fig. S2, the structural differences for the two studies are as follows:

- Different normalization and denormalization: min-max normalization and denormalization for linear-array probe study, z-score normalization and denormalization for convex-array probe study.
- Different activation functions in the last transposed convolutional block: a Tanh activation for the linear-array probe study, and no activation function for the convex-array probe study.

**Fig. S2. Details of the Extrapolation Models for (a) Linear- and (b) Convex-array Probe Studies.^a^ The numbers above each rectangle indicate the number of filters. Red arrows highlight structural differences between (a) and (b).**

**^a^Abbreviations: min-max normalization: the sinogram is mapped to [-1, 1] from the probe’s value range of [-32768, 32768]; min-max denormalization: the inverse calculation of min-max normalization. Other abbreviations are the same as those in Figs. 1 and 2.**

**(2) Reasons for Structural Differences Between the Two Studies**

The linear-array probe study serves as our main study. In this study, the samples cover a signal range with relatively small variance (as shown in Fig. S3(b)). Therefore, min–max normalization was applied to constrain the data within the probe value range of [-32768, 32768]. Accordingly, a Tanh activation function, which maps values to [-1, 1], was used in the final layer to ensure that the model outputs remain within the valid range.

When transferring the extrapolation model to the convex-array probe study, we found that the samples exhibit a large signal variance, leading to inconsistent data distributions across samples (see the first column in Fig. S3(a)). In this case, applying min–max normalization as in the linear-array study would harm the model’s generalization ability due to the substantial discrepancy in data distributions (see the second column in Fig. S3(a)). Therefore, we adopted z-score normalization to standardize each sample to zero mean and unit variance (see the similar distributions in the third column of Fig. S3(a)). Correspondingly, the Tanh activation used in the linear-array study is no longer suitable under this normalization scheme and was therefore removed.

**Fig. S3. Kernel Density Estimates of Representative Data from the (a) Convex-array and (b) Linear-array Probe Studies under the Original Signal Domain and Different Normalization Strategies.**

**(3) Analysis of Differences in Loss Functions Between the Two Studies**

In the main manuscript, Eqs. (1) and (2) represent the loss functions used in the linear- and convex-array probe studies, respectively, for training extrapolation models. Eq. (1) refers to a weighted mean square error (MSE), which retains the original signal values due to the relatively small variation in their ranges (as shown in the first column in Fig. S3(b)). In contrast, Eq. (2) adopts a relative root mean square error (rRMSE), which normalizes the signal values to account for the large discrepancies in magnitude (see the three-orders-of-magnitude difference in the first column in Fig. S3(a)). If the same loss function as in Eq. (1) was used for the convex-array probe study, samples with large values would dominate the training process, potentially neglecting errors associated with small-valued samples.

**S3. Discussion about the Design of Our Extrapolation Model**

In our extrapolation model, we proposed (a) the RotDRConv and RotTransDRConv to approximate the signal variations caused by rotation, and (b) the rotation angle *γ* to be -60°, -24°, 24° and 60°. In the following subsections, we give a detailed discussion of the reasons for such designs.

**(1) Details of the Proposed RotDRConv and RotTransDRConv**

Fig. 3(b) shows the structures of RotDRConv and RotTransDRConv. Fig. S4 provides further details of these two self-designed convolution modules.

**Fig. S4. Details of the Proposed RotDRConv and RotTransDRConv.^a^ RotDRConv and RotTransDRConv share the same architecture but differ in the convolution type, as indicated by the red “Conv” in the figure.**

**^a^Abbreviations: *n*, filter number; *N*, filter number of RotDRConv or RotTransDRConv; conv, convolution; transposed conv, transposed convolution.**

**(2) The Reason for the Proposed RotDRConv and RotTransDRConv**

We designed the RotDRConv and RotTransDRConv to fit the rotation-induced signal changes. There are two points about such signal change: (1) for one ultrasound probe consisting of multiple transducer elements, as shown in Fig S5(a), the rotation-induced signal change received by different transducer elements would be diverse; (2) the rotation-induced signal change received by the same transducer is discrepant because of the various rotation angles, as shown in Fig S5(b).

**Fig. S5. Illustration for Why the RotDRConv and RotTransDRConv Fit the Rotation-induced Signal Change. (a~b) illustrate 2 points about the rotation-induced signal variation. (c) shows the architectures of RotDRConv and RotTransDRConv and their design points for (a-b). ^a^**

**^a^The abbreviations in (c) are the same as those in Figs. 3 and S4.**

For point (1), we adopt the DRConv^1^, since DRConv would use different convolution kernels according to its classifications. For the DRConv proposed by Chen et al.^1^, the classifications are learnable. In our RotDRConv and RotTransDRConv, the classifications are evenly specified according to the multiple transducers’ spatial positions. For point (2), we employ fully-connected layers to generate kernel-regulating parameters (i.e., *R* in Fig S5(c)) according to the rotation angle *γ*. The difference between RotDRConv and RotTransDRConv is that they adopt different convolutions. The former one uses a convolution. The latter one uses a transposed convolution^2^.

**(3) The Reason for the Values of *γ***

Our work limits the *γ*’s value in the range of [-60°, -24°, 24°, 60°]. There are two reasons.

The first reason is that, in our EXPP-Net, the extrapolation model’s purpose is to generate the sinograms at other angles to reduce the image distortion caused by single view acquisition, and hence to decrease the task difficulty of the post-processing model. Therefore, the *γ*’s values should be evenly distributed over the remaining angles, and we chose 24° and 60° as the two rotation angles.

**Fig S6. Illustration for why the *γ*^a^∈(-90°, 90°)**

**^a^The *γ* shares the same meaning in Fig 3(a)**

The second reason is that the convolution inherence limits *γ*∈(-90°, 90°). As shown in Fig. S6(a), the convolution’s inherent calculation is that the output y*_ij_* (*i* and *j* represent the pixel position in one image) is the computation result of the input image’s pixel values within a specific patch. The patch’s center is (*i*, *j*). When *γ* falls outside the range of (-90°, 90°), the signal from the same object would be received by the transducer at the opposite end of the probe (as the *m_α_*_+_*_γ_*_2_ in Fig. S6(b)). This is not an applicable scenario for a convolution as shown in Fig. S6(c).

**S4. Electrical Excitation Parameters across All Studies**

Table S2 lists the electrical excitation parameters for each sample in all studies in this manuscript.

**Table S2. Summary of Electrical Excitation Parameters for All Studies**

| **Study type** | **Data source** | **Sample No.** | **Electrical pulse** | | | **Distance between electrodes (mm)** | **Electric Field Strength^a^ (Volts/cm)** |
| --- | --- | --- | --- | --- | --- | --- | --- |
|  |  |  | **Width (ns)** | **Amplitude (kV)** | **Repetition frequency (Hz)** |  |  |
| Linear-array probe study | Water tank | 1~18 | 100 | 0.8 | 20 | 5 | 1600 |
|  |  | 19~30 | 100 | 0.6 | 20 | 5 | 1200 |
|  |  | 31~44 | 100 | 1 | 20 | 5 | 2000 |
|  |  | 45~54 | 100 | 1.2 | 20 | 5 | 2400 |
|  | mouse | 1 | 100 | 2 | 20 | 5 | 4000 |
| Convex-array probe study | Water tank | 1~4 | 100 | 1.0 | 50 | 5 | 2000 |
|  |  | 5~7 | 100 | 1.5 | 50 | 5 | 3000 |
|  |  | 8~11 | 100 | 2.0 | 50 | 5 | 4000 |
|  |  | 12~15 | 100 | 2.5 | 50 | 5 | 5000 |
|  |  | 16~19 | 100 | 3.0 | 50 | 5 | 6000 |
|  |  | 20~21 | 100 | 3.5 | 50 | 5 | 7000 |
|  |  | 22 | 100 | 4.0 | 50 | 5 | 8000 |

^a^ Electric Field Strength = Amplitude/Distance between electrodes.

**S5. Probe-electrode Geometry**

As shown in Fig. S7, the electrode pair is positioned within the ultrasound imaging region at varying distances from the probe (see Fig. S7 (a)). The imaging plane is perpendicular to the longitudinal axes of the needle electrodes (see Fig. S7 (b)). Fig. S7 takes a convex-array probe as an example, while the study on the linear-array probe employs the same geometry as shown in Fig. S7.

**Fig S7. Illustration of the Probe-electrode Geometry. ^a^**

^a^Abbreviations: *d*: the distance from the probe center to the electrode pair center; *θ*: angle between the axis from the electrode pair center to the probe center and the central axis of the probe (positive if electrode pair center is above the central axis of probe; negative below). The value ranges of *d* and *θ* are listed in Table S3. The linear-array probe study shares the same geometry.

To further clarify our experimental setup, we define the distance from the probe center to the electrode pair center (denoted as *d* in Fig. S7 (a)) and the angle between the axis from the electrode pair center to the probe center and the central axis of the probe (denoted as *θ* in Fig. S7 (a)). The value ranges of these two notations are listed in Table S3.

**^Table S3. Probe–Electrode Geometrya^**

| **Study Type** | ***d* (mm)** | ***θ* (°)^b^** |
| --- | --- | --- |
| **Linear-array probe study on water-tank data** | 25.73~44.01 | -16.17~18.29 |
| **Convex-array probe study on water tank data** | 63.22~80.89 | -11.62~7.88 |

^a^Abbreviations: *d*, the distance from the probe center to the electrode pair center; *θ*, the angle between the axis from the electrode pair center to the probe center and the central axis of the probe. A schematic illustration of *d* and *θ* is shown in Fig. S7.

^b^Positive *θ* indicates the electrode pair center is above the probe’s central axis (as shown in Fig. S7(a)); negative *θ* indicates it is below.

**S6. Ultrasound Data Acquisition and Processing**

This section provides additional details on ultrasound data acquisition and processing.

**(1) Ultrasound Acquisition Without Compounding**

No conventional ultrasound compounding was applied in this study. The probe operated in a passive receiving mode, capturing acoustic signals generated by the electrodes. Multi-angle measurements were not fused into a compounded image; instead, they were directly used as input to a tomographic reconstruction algorithm (i.e., back projection).

**(2) Signal Filtering and Image Resolution**

During data acquisition, a hardware band-pass filter (150kHz-15Mhz) was applied to the signals. No additional filtering was performed during data processing; the raw signals were directly used for reconstruction with an image resolution of 0.1mm/pixel.

**(3) Effects of Reflected Echoes from Tank Walls**

The water tank is sufficiently large (Depth×Width×Height: 12 inches×12 inches×6 inches), and the electrodes and probe were positioned to minimize reflections from the tank walls. Therefore, any echo contribution from the boundaries is expected to be negligible.

**S7. Ground Truth Construction Workflow**

To get a full-view image as ground truth, each single-view sinogram was first reconstructed into a single-view image using the back-projection (BP) algorithm without any filtering. The reconstructed images were then rotated according to their corresponding acquisition angles and aligned at their rotation center. Finally, all rotated images were averaged pixel-wise to generate the full-view image. No additional post-processing was applied.

The rotation center (denoted as (*x*_o_, *y*_o_)) was calculated using the following formula:

in which (*x*_1_, *y*_1_) and (*x*_2_, *y*_2_) denote the coordinates of the maximum intensity point of the same electrode in the reconstructed single-view images acquired at angles *α* and *β*. (*x*_1_, *y*_1_) and (*x*_2_, *y*_2_) were manually determined using the *getpts* function in MATLAB, which provides the coordinates of user-selected points via mouse clicks.

**S8. Strategies for Mitigating Overfitting and Underfitting**

In this section, we describe the strategies used to mitigate overfitting and underfitting, supported by relevant references and experimental results.

**(1) Strategies for Mitigating Overfitting and Supporting Results**

To mitigate overfitting, a validation set disjoint from the training set was employed to monitor the training process. The model achieving the lowest validation loss was selected as the final model, as suggested by Ian Goodfellow et al.^3^.

Mechanism:

Overfitting occurs when a model fails to generalize to unseen data. Using a validation set which is disjoint from training data and selecting the model with the lowest validation loss helps mitigate overfitting by promoting better generalization.

Supporting Results:

To further evaluate the overfitting mitigation performance, we illustrate training and validation losses over epochs in Fig. S8. The training and validation losses decrease consistently without a significant gap, indicating that the model does not suffer from overfitting.

**Fig. S8. Training and Validation Loss Curves of EXPP-Net: (a) Extrapolation Model and (b) Post-processing Model.**

**(2) Methods to Mitigate Underfitting and Supporting Results**

To diagnose underfitting, we (a) monitored both training and validation losses throughout the training process^3^. A high loss on both the training and validation sets was used as an indicator of underfitting. In addition, (b) a ReduceLROnPlateau (Reduce Learning Rate on Plateau) learning rate scheduling strategy^4,5^ was applied to improve optimization and convergence during training. Although these techniques do not directly prevent underfitting, they help identify suboptimal learning behavior. When underfitting was observed, model capacity was increased by using deeper architectures or by extending the training duration.

Mechanism:

Underﬁtting occurs when the model is not able to obtain a suﬃciently low error value on the training set. Therefore, displaying training loss over epoch (above method (a)) is a direct way to diagnose underfitting.

The ReduceLROnPlateau strategy is used to facilitate convergence during training. It reduces the learning rate when the training loss stops improving, allowing finer parameter updates in later stages. This helps stabilize optimization and improve convergence.

By adaptively reducing the learning rate on plateaus, this strategy also reduces the risk of misinterpreting slow convergence as underfitting during early or intermediate training stages.

Supporting Results:

To evaluate whether underfitting occurs, we monitored the training loss throughout the optimization process. As shown in Fig. S8, the training loss decreases steadily and converges to a low stable value, indicating that the model successfully captures the underlying patterns in the training data. Although the loss does not reach zero due to inherent noise and problem complexity, this does not indicate underfitting. In addition, the validation loss follows a similar trend, further confirming that the model is not underfitting.

**S9. Benchmark Selection Rationale and Technical Comparison with EXPP-Net**

**(1) Benchmark Selection Rationale**

There are two main reasons. (a) This benchmark addresses the same problem as ours, namely single-view artifact correction for monitoring electroporation using ultrasound imaging (as shown in Fig. S9), ensuring a fair and task-relevant comparison. (b) It is built upon the widely adopted U-Net architecture, which has demonstrated strong and reliable performance across a variety of related artifact correction tasks, and is commonly used as a competitive baseline in the literature^6–9^.

**Fig. S9.** **Illustration of Single-view Artifacts. The intensity discrepancies between single-view and full-view images indicate artifacts. The white dashed boxes show representative examples, while similar artifacts are present elsewhere in the image.**

**(2) Technical Comparison Between the Benchmark Strategy and EXPP-Net**

Both the benchmark and our EXPP-Net employ image-domain enhancement networks. However, the benchmark applies enhancement directly to the input images, despite the presence of varying distortion patterns (referred to as “Direct Enhancement” in Fig. S1). In contrast, as shown in the “Preconditioned Enhancement” panel of Fig. S1, our EXPP-Net first utilizes an extrapolation network to generate four additional sinograms, which are then reconstructed together with the original single-view sinogram into a five-view image. This process results in a more uniform distortion pattern, thereby simplifying the subsequent enhancement task. A schematic comparison of the two methods is illustrated in Fig. S1.

**References:**

1.	Chen J, Wang X, Guo Z, Zhang X, Sun J. Dynamic Region-Aware Convolution. *arXiv*. Preprint posted online March 15, 2021:arXiv:2003.12243. doi:10.48550/arXiv.2003.12243

2.	Zeiler MD, Taylor GW, Fergus R. Adaptive deconvolutional networks for mid and high level feature learning. In: *2011 International Conference on Computer Vision*. IEEE; 2011:2018-2025. doi:10.1109/ICCV.2011.6126474

3.	Goodfellow I, Bengio Y, Courville A. *Deep Learning*. The MIT Press; 2016.

4.	Anwar T, Zakir S. Deep learning based diagnosis of COVID-19 using chest CT-scan images. In: *2020 IEEE 23rd International Multitopic Conference (INMIC)*. IEEE; 2020:1-5. doi:10.1109/INMIC50486.2020.9318212

5.	You K, Long M, Wang J, Jordan MI. How Does Learning Rate Decay Help Modern Neural Networks? *arXiv*. Preprint posted online 2019. doi:10.48550/ARXIV.1908.01878

6.	Zhang R, Wang J, Wang L, Deng B, Hu J. Synchrotron based sparse-view CT artifact correction with STC-UNet. *Nucl Instrum Methods Phys Res Sect Accel Spectrometers Detect Assoc Equip*. 2025;1073:170306. doi:10.1016/j.nima.2025.170306

7.	Lu K, Zhang Z, Ren L, Yin F. Deep residual network‐based projection interpolation and post‐processing techniques for thoracic patient CBCT reconstruction. *Med Phys*. 2025;52(7):e17953. doi:10.1002/mp.17953

8.	Huang M, Li G, Sun R, et al. Sparse-View Artifact Correction of High-Pixel-Number Synchrotron Radiation CT. *Appl Sci*. 2024;14(8):3397. doi:10.3390/app14083397

9.	Chan Y, Liu X, Wang T, Dai J, Xie Y, Liang X. An attention-based deep convolutional neural network for ultra-sparse-view CT reconstruction. *Comput Biol Med*. 2023;161:106888. doi:10.1016/j.compbiomed.2023.106888
